# An antibody uniquely binding short 2′-O-methyl RNA oligonucleotide duplexes: formation and recognition of target duplexes on cell surfaces

**DOI:** 10.3389/fimmu.2025.1699400

**Published:** 2026-01-12

**Authors:** Ian S. Dunn, Matthew M. Lawler, Leah Fagundes, Milto Simoes Junior, April Mangold, Domenic Rinaldi, Lenora B. Rose, James T. Kurnick

**Affiliations:** 1 TriBiotica LLC, Quincy, MA, United States; 2 Department of Pathology, Massachusetts General Hospital, Boston, MA, United States

**Keywords:** 2’-O-methyl RNA binding, cancer diagnosis and therapy, cell surface targets, short duplex recognition, surface annealing, unique monoclonal antibody

## Abstract

With the primary aim of generating antibody tools useful for diagnostic and therapeutic applications relevant to oligonucleotide-templated reactions, an scFv filamentous phage library was screened using a specific 2′-O-methyl RNA template:oligonucleotide click reaction complex as the target structure. Such an antibody would not be expected to recognize any naturally occurring biological molecules. Two promising candidate scFv clones were converted into human IgG1 antibodies for further characterization. Surprisingly, although the best antibody (IgG1-DS5) was bound to the original selection complex, its recognition preference was shown to be directed toward short 2′-O-methyl RNA duplexes without click modifications. The IgG1-DS5 antibody showed no binding to the separate single strands comprising the recognized duplex, nor to the corresponding duplexes with RNA or DNA template strands. Versions of the target 2′-O-methyl RNA duplex with divergent sequences were also not recognized by IgG1-DS5. The unexpected binding properties of the IgG1-DS5 antibody were directed toward potential applications based on the molecular proximity of tethered single strands. Initially, SKBr3 cells were coated with biotins by means of surface azide metabolic labeling with peracetylated N-azidoacetylmannosamine, followed by treatment with a click-reactive DBCO-PEG4-biotin compound. Subsequent cell treatment with the tetravalent biotin-binding protein neutravidin (NAV), carrying subsaturating levels of biotinylated 2′-O-methyl RNA target duplexes, showed strong IgG1-DS5 staining on cell surfaces. These observations were extended with biotinylated anti-EGFR antibody linked with biotinylated 2′-O-methyl RNA single strands, also by means of the NAV protein as an adaptor. Flow cytometry analysis showed that DS5 antibody binding was only obtained when combinations of separate preparations of antibodies carrying top and bottom target strands were applied sequentially to EGFR-positive cells. These results show that proximity-based surface annealing of the IgG1-DS5 antibody target single strands can act to define cell populations with a surface marker of sufficient density. Where IgG1-DS5 is derivatized with either a fluorescent moiety or a cytotoxic drug, this antibody may find application in diagnostic or therapeutic tumor targeting.

## Introduction

Nucleic acid-templated synthesis and proximity focusing have had numerous applications in biological research and biotechnology (1–5). We have previously studied systems where short oligonucleotides modified with reactive moieties (“haplomers”) can be assembled via a mutually complementary template (6). As an extension of this work, we reasoned that an antibody capable of specifically recognizing a templated click reaction product would be useful for general reactivity studies and cell targeting. Accordingly, for the antibody target, we used short haplomers comprised of 2′-O-methyl oligonucleotides modified with 5′ bicyclononyne (BCN) or 3′-azide moieties, annealed in proximity with a mutually complementary template. As this target structure is absent from known molecular repertoires of living organisms, an antibody binding it would not be anticipated to recognize any natural biological molecules (a bioorthogonal specificity).

This structure then acted as a selection target for panning with a large scFv filamentous phage library (7). A panel of scFv candidate proteins was screened for selective recognition of the haplomer:template complex over template alone, and coding sequences for the V_H_ and V_L_ segments from the most promising scFv candidates were subsequently fused with appropriate immunoglobulin heavy chain and kappa light chain C-region sequences to convert them into expressible complete human IgG1 molecules. The best resulting candidate is referred to here as IgG1-DS5.

During functional recognition tests for IgG1-DS5 in ELISA assays, it was discovered that this antibody was surprisingly not dependent on the BCN or azide oligonucleotide modifications for target binding. Moreover, strong binding signals were obtained with 25-mer or 22-mer 2′-O-methyl RNAs (corresponding to the 5′-region of the original template) duplexed near their 3′-end with a 9-mer 2′-O-methyl RNA. The binding specificity was remarkably specific toward 2′-O-methyl RNAs, in that IgG1-DS5 showed no recognition toward corresponding duplexes with RNA or DNA templates. Duplexes of 2′-O-methyl RNAs with unrelated sequences to the original target were also not bound by IgG1-DS5. This recognition pattern serendipitously lent itself toward certain novel applications of the unique IgG1-DS5 antibody, directed toward a bioorthogonal and nuclease-resistant structure.

Assays for markers on cell surfaces in close molecular proximity have numerous applications, including diagnostic and therapeutic options for tumor targeting (8–10). When tagged with oligonucleotides, antibodies directed against markers of interest can be exploited for proximity assays. When brought sufficiently close, the antibody-appended oligonucleotides can be ligated together or serve to ligate a third-party oligonucleotide, either of which can then act as a substrate for an amplification process for identifying positive signals (11, 12). This “proximity ligation assay” (PLA) has had widespread use as a sensitive means for the *in vitro* characterization of cell surface marker proximity in various instances (13–16). While effective in this regard, implementation of the PLA is complex and cannot be applied to *in vivo* situations. The unique binding specificity of IgG1-DS5, as demonstrated here, is directly relevant to proximity assays either *in vitro* or *in vivo*. If antibodies carrying single-stranded IgG1-DS5 target oligonucleotides bind to spatially proximal targets, there is an opportunity for the complementary single strands to form duplexes, which in turn become targets for IgG1-DS5 recognition. Since no binding of single strands occurs, signals arising from IgG1-DS5 in such circumstances serve as accurate gauges of the molecular proximity of the markers targeted by the antibodies used in the specific assay. Given the experimentally determined bioorthogonal specificity of IgG1-DS5, this antibody has wide potential application toward *in vivo* targets, including conveying fluorescent or radioisotopic tags for diagnostic purposes or cytotoxic drugs for antitumor therapeutic modalities. This is particularly relevant for recent efforts toward identifying multiple cell surface markers for coordinated tumor targeting, given the relative paucity of single markers that can distinguish tumors from normal cells (9, 10).

As a facile alternative to chemical or other conjugation methods for tagging with relevant oligonucleotides, here, we have used biotinylation of both primary antibodies and single 2′-O-methyl RNA strands in order to use a biotin-binding protein [neutravidin (NAV)] as an adaptor for oligonucleotide–antibody linkage. This approach also permits the use of metabolic labeling (17, 18) to coat cell surfaces with biotins in order to assess the direct recognition of duplexes. When bound to surface markers in proximity, antibodies tagged with single strands (via NAV) can permit the formation of duplex targets for IgG1-DS5 binding. In this manner, either the same marker in high density or two separate markers can be used to distinguish tumor cells from normal by means of their surface phenotypes.

## Materials and methods

### Selection of scFvs binding the 2′-O-methyl RNA template/haplomer system and conversion to human IgG1 antibody

**Figure 1:**
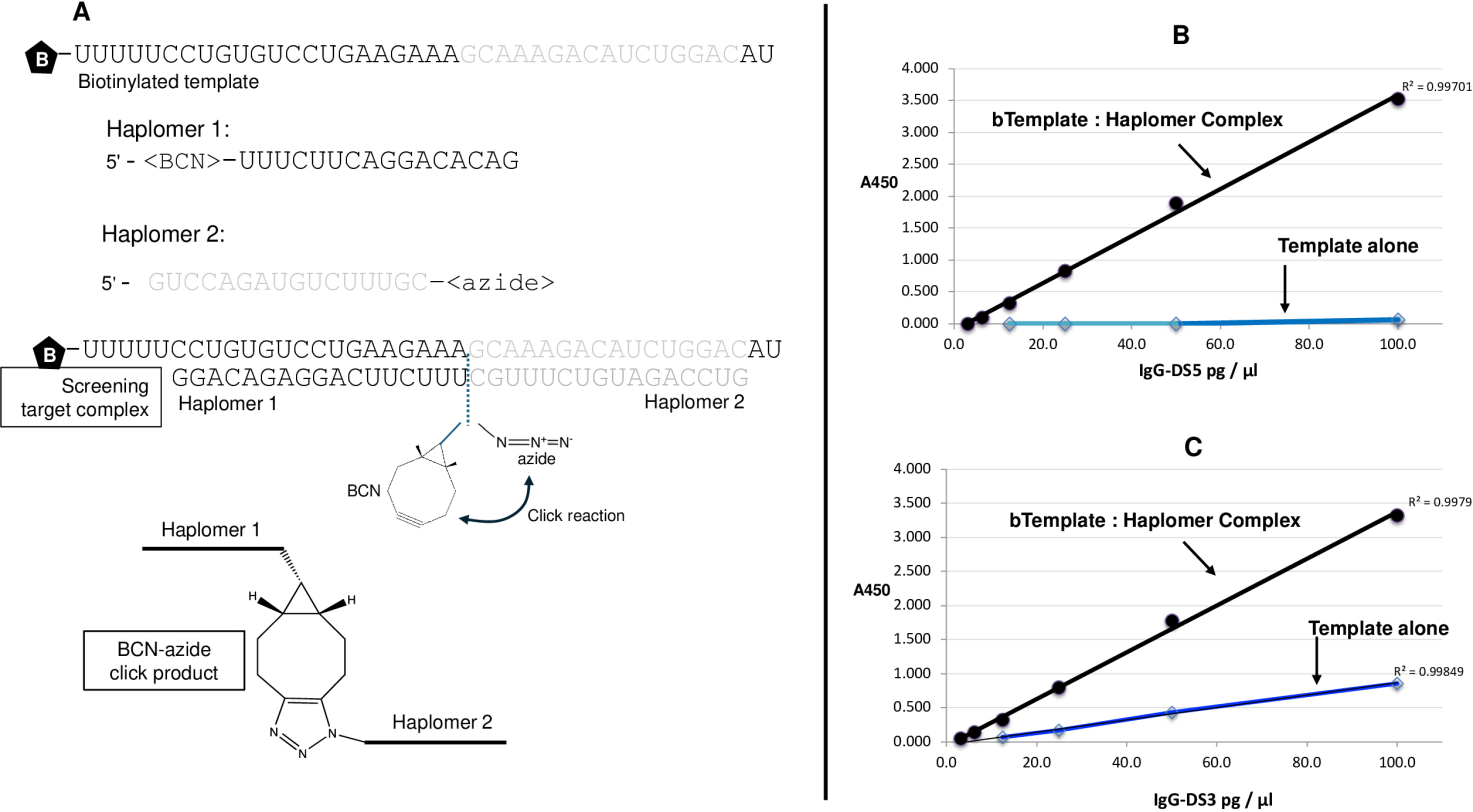
**(A)** Sequences and functions of the original modified 2′-O-methyl RNA oligonucleotides used for selection for scFv binders from a large phage library. The oligo (haplomer) modifications with bicyclononyne (BCN; haplomer-1) or azide (haplomer-2) are as shown. When brought into proximity by the mutually complementary template, the BCN and azide moieties react to form the indicated triazole reaction product. **(B)** ELISA screening of candidate antibodies IgG1-DS5 **(B)** and IgG1-DS3 **(C)** with preformed template:haplomer complexes **(A)** or template alone. Correlation coefficients for linear-range ELISA responses shown as *r*^2^ values.

The DS5 antibody specificity was originally selected from a large phage scFv library (Creative Biolabs, Shirley, NY, USA; human HuScL-6 filamentous phage library, capacity 10^11^ clones) against a templated oligonucleotide complex, where all components were 2′-O-methyl RNAs, and with the short oligonucleotides (“haplomers” (6);) chemically modified with click-reactive BCN and azide moieties (BioSynthesis; **Figure 1**). When assembled onto the longer mutually complementary template oligonucleotide, the haplomer chemical modifications react to form a triazole product (**Figure 1**). The longer template strand was biotinylated at its 5′-end to allow binding of the entire complex to solid-phase streptavidin and thus panning cycles for binding selection with the phage library. Panning methods were based on well-established previous technology (19). Since the original desired binding specificity was toward the central click product (with or without specificity for flanking 2′-O-methyl RNA residues), enriched binding populations were negatively selected against solid-phase biotinylated template alone or biotinylated versions of haplomer-1 and haplomer-2 (**Figure 1**; biotinylated at the 3′- and 5′-ends, respectively).

Candidate scFv binders were selected by a proprietary affinity/clone sequence frequency-based method (Creative Biolabs, Shirley, NY, USA; Magic™ Therapeutic Antibody Discovery Platform) and screened in ELISA assays for binding to the desired complex vs. undesired binding to template alone or haplomer strands alone. Two promising candidates were then converted into human IgG1 by complete synthesis of sequences of the specific V_H_ regions joined in frame with human γ1 C-region coding sequence and V_L_ regions with human κ light chain sequence. Full coding sequences were cloned into the expression vector pcDNA3.1 and expressed and purified from HEK 293F cells (Creative Biolabs, Shirley, NY, USA).

### Oligonucleotide syntheses

Oligonucleotides were synthesized by IDT (Coralville, IA) or BioSynthesis Inc. (Lewisville, TX). Oligonucleotides used in this study are shown in **Table 1**.

**Table 1:** Oligonucleotides used in this study.

Oligonucleotide name	<Modifications>/sequence 5′–3′	Backbone
Temp1	<biotin>-UUUUUCCUGUGUCCUGAAGAAAGCAAAGACAUCUGGACAU	2′-O-methyl RNA
Haplomer-1	<BCN>-UUUCUUCAGGACACAG	(“ “ “)
Haplomer-2	GUCCAGAUGUCUUUGC–<azide>	(“ “ “)
Oligo-1	UUUCUUCAGGACACAG	(“ “ “)
Olig1Ext1	UUUCUUCAGGACACAGGUUUUU	(“ “ “)
Olig1Ext2	UUUCUUCAGGACACAGGAAAAA	(“ “ “)
Oligo-2	GUCCAGAUGUCUUUGC	(“ “ “)
Contig. complement-1	AUGUCUUUGCUUUCUUCAGG	(“ “ “)
Contig. complement-2	GUCCAGAUGUCUUUGCUUUCUUCAGGACACAG	(“ “ “)
9-co	UUUCUUCAG	(“ “ “)
S9-co	<biotin>-Sp18-UUUCUUCAG *	(“ “ “)
Temp-F	UUUUUCCUGUGUCCUGAAGAAAGCAAAGACAUCUGGACAA-<FAM>	(“ “ “)
Temp2O	UUUUUCCUGUGUCCUGAAGAAAGCAAAGACAUCUGGACAA	(“ “ “)
TempTr1	<biotin>-UUUUUCCUGUGUCCUGAAGAAAGCA	(“ “ “)
S-Tr1	<biotin>-Sp18-UUUUUCCUGUGUCCUGAAGAAAGCA *	(“ “ “)
Tr1-Ext1	UUUUUCCUGUGUCCUGAAGAAAGCAAAGACA	(“ “ “)
Tr1-Ext2	UUUUUCCUGUGUCCUGAAGAAAGCAAAGACAUCU	(“ “ “)
TempTr2	<biotin>-UUUUUCCUGUGUCCUGAAGAAA	(“ “ “)
TempTr3	<biotin>-UUUUUCUGAAGAA	(“ “ “)
TempTr4	<biotin>-UUUUUCUCUCUGGCUGAAGAAA	2′-O-methyl RNA
TempTr5	<biotin>-UUUUUCGUGUGUGUCUGAAGAAA	(“ “ “)
TempTr6	<biotin>-UUUUUCGUGUGUCCUGAAGAAA	(“ “ “)
TempTr7	<biotin>-UUUUUCGUGUGUGCUGAAGAAA	(“ “ “)
TempTr8	<biotin>-UUUUUCCUGUGUCACAAUAGAG	(“ “ “)
TempFlp	<biotin>-UUUCUGAAGAAAUUUCCUGUGUC	(“ “ “)
ALT-M1	<biotin>-GUAACUGGUUCUUUCAGAAUUAUCAGUGAGCCAACGCAUA	(“ “ “)
ALT-M2	<biotin>-GGAAUCUGUUCUUUCAGAAUUAUCAGUGAGCCAACGCAUA	(“ “ “)
ALT-haplomer-1	<BCN>-UAAUUCUGAAAGAACC	(“ “ “)
Oligo-3	UAAUUCUGAAAGAACC	(“ “ “)
ALT-haplomer-2	UGCGUUGGCUCACUGA–<azide>	(“ “ “)
Oligo-4	UGCGUUGGCUCACUGA	(“ “ “)
FAM-probe1	<FAM>-TTTCTTCAGGACACAG	DNA
FAM-probe2	<FAM>-TGTCCAGATGTCTTTGC	DNA

*Sp18=(PEG)6 spacer (IDT).

### Cell lines

SKBr3 and SiHa cells were both obtained from the American Type Culture Collection (ATCC), University Boulevard Manassas, VA, USA.

### ELISAs for IgG1-DS3 and IgG1-DS5 antibody characterization

For ELISA analyses, oligonucleotides to be tested for antibody binding were immobilized in 96-well plates, usually via biotinylation of one oligonucleotide strand for binding to wells of streptavidin (SA) plates (R&D Systems, Minneapolis, MN, USA). An alternative used in some situations was via anti-FAM antibodies (Rockland Immunochemicals, Pottstown, PA, USA) and terminal labeling of one oligonucleotide strand with a FAM moiety (IDT). The anti-FAM antibody was immobilized on standard ELISA plates at a concentration of 2 µg/mL in sodium bicarbonate buffer, pH 9.0 (100 µL/well) for 16 h at 4°C, before blocking the plate with 10 mg/mL of bovine serum albumin (BSA, Sigma, Saint Louis, MO, USA; 200 µL/well) for another 16 h at 4°C. Labeled oligonucleotides were captured in plate wells at 0.5 pmol/µL. Washing steps between sequential additions of reagents were performed with 200 µL/well of phosphate-buffered saline (PBS)/0.05% Tween 20. To reduce background binding of antibodies, blocking agents were important. PBS with 1% fatty acid-free BSA (Sigma, Saint Louis, MO, USA) was suitable, but the best and most cost-effective blocking reagent proved to be PBS with 1.25% Blotto (powdered milk). The secondary antibody for the human antibodies IgG1-DS3 and IgG1-DS5 was goat anti-human kappa chain, horse radish peroxidase (HRP) conjugate. Final development of signals from ELISA wells was via the standard TMB reagent (BioLegend, San Diego, CA, USA), with subsequent colorimetric plate reads at 450 nM after stopping the reactions with 1 M of sulfuric acid.

### Gel-shift assays for antibody binding and duplex analysis

Gel (mobility)-shift assays for antibody binding of templated complexes were performed with test single-stranded or duplex oligonucleotides (20 µM) and IgG1-DS5 antibody (25 ng/µL final concentration) in PBS for 1 h/room temperature, before running samples on 5% Tris/glycine (TG) gels and final staining with SYBR-Gold. Assays for duplex formation of short oligonucleotides were performed in a similar manner, but with 40 µM of oligonucleotides to enhance band visibility after staining, and 10%–15% TG gels were used that were supplemented with 10 mM of magnesium chloride and 10 mM of potassium acetate.

### SPR analyses

Surface plasmon resonance (SPR) measurements for IgG1-DS5 were conducted by Olympic Protein Technologies, Seattle, WA. Oligonucleotide analytes were synthesized by IDT and were pure as assessed using sensitive SYBR-Gold staining by gel electrophoresis. The data were aligned, double-referenced, and fitted using Scrubber v2.0^©^ software (BioLogic Software Pty Ltd, Campbell, Australia), an SPR data processing and non-linear least squares regression fitting program. The average association phase binding data from 110 (s) to 115 (s) for each duplicate concentration were used to produce the response to concentration binding plots. The binding plots were analyzed by globally fitting the data to a single-site interaction model to determine the equilibrium dissociation constant (*K*_D_) and the *R*_max_ values.

### Use of biotin-binding neutravidin as an adaptor in proximity assays by flow analysis

The tetravalent biotin-binding protein NAV (a deglycosylated version of avidin with a pI close to neutrality, Thermo Fisher Scientific, Waltham, MA, USA) can be used as an adaptor for coupling target duplex or single-stranded antibodies to biotinylated primary antibodies. In the most effective process, biotinylated anti-EGFR or anti-HER2 (R&D Systems, Minneapolis, MN, USA) was incubated with a theoretical 2.5-fold excess of NAV (based on an average biotinylation ratio of 10 biotins/antibody molecule; R&D Systems, Minneapolis, MN, USA) for 30 min at room temperature, followed by the addition of a 40-fold superexcess of the biotinylated single strand or duplex of interest for another 30 min at room temperature. Cells of interest (bearing surface markers recognized by the antibodies for the assay) were then incubated with the antibody/NAV/oligonucleotide complexes for a single 45-min incubation on ice for preformed target duplexes. Where surface annealing of separate strands was desired, antibodies bearing each strand (via NAV adaptation) were separately annealed in two steps of 30 min on ice, with a washing step between antibody-strand additions. Following the final incubations, cells were treated with IgG1-DS5 or an antibody against NAV (Rockland Immunochemicals, Pottstown, PA, USA), each at 2 µg/mL final, for 30 min on ice. After washing in PBS, cells were finally treated with secondary goat-anti-human kappa FITC (for IgG1-DS5 probings) or goat anti-rabbit-FITC (for anti-NAV antibody), before standard flow analysis.

### Metabolic labeling for surface placement of biotin, biotin-binding proteins, and biotinylated oligonucleotides

To generate surface azide moieties as terminal modified sialate residues, cells of interest were treated with 100 µM of peracetylated N-azidoacetylmannosamine (AzNAM, Sigma, Saint Louis, MO, USA) in 6-well plates for 24 h, before harvesting. After adjustment to 10^6^/mL in serum-free RPMI medium with 0.5% fatty acid-free BSA (SFRP-FBSA; Sigma, Saint Louis, MO, USA), cells were treated with or without dibenzocyclooctyne (DBCO)-PEG4-biotin (DPEG-B, 20 µM final, BroadPharm, San Diego, CA, USA) for 1 h at room temperature, with frequent resuspensions. Following such treatments, cells were pelleted and washed 1× with SFRP-FBSA, and finally 1× with PBS alone. NAV bearing biotinylated single strands or duplexes was initially bound with a 1.5–2.5 molar excess of oligonucleotides for 1 h at room temperature in PBS, before adding to AzNAM/DPEG-B-treated cells or controls prepared as above. Following the final incubations, cells were treated with IgG1-DS5 or an antibody against NAV (each at 2 µg/mL final) for 30 min on ice. After washing in PBS, cells were finally treated with secondary goat-anti-human kappa FITC (for IgG1-DS5 probings) or goat anti-rabbit-FITC (for anti-NAV antibody), before standard flow analysis.

### Flow statistical analyses

Flow data (peak geometric means with standard deviations and gated numbers of events) were analyzed using GraphPad software to perform unpaired one-tailed Student’s ***t***-tests.

## Results

### Candidate antibody binding of the template vs. template:haplomer complex

The two candidate antibodies derived from the scFv phage library screening (IgG1-DS5 and IgG1-DS3; Methods) were initially tested for binding activity toward the full template: BCN- and azide-haplomer complex (**Figure 1A**) vs. template alone. In ELISA assays with streptavidin plates used to immobilize biotinylated oligonucleotide strands and hybridized complements (Methods, ELISAs), it was found that IgG1-DS5 strongly recognized the complete template:BCN-/azide-haplomer complex while unresponsive toward template alone, whereas IgG1-DS3 consistently showed a lower but definite binding to template without the hybridized haplomers (**Figures 1B,C**). Reactions of IgG1-DS5 could be obtained in ELISA assays equally well with either preformed biotinylated template:haplomers or by plating of biotinylated template, followed by sequential hybridizations with azide-haplomer followed by BCN-haplomer. No reactivity was seen toward biotinylated BCN-haplomer plated with azide-haplomer in the absence of template (**Supplementary Data**, **Supplementary Figure S1**). Since IgG1-DS3 failed to show the desired degree of specificity toward the template:haplomer complex, all subsequent functional studies favored its counterpart antibody IgG1-DS5.

### Strand specificity of IgG1-DS5 recognition

**Figure 2:**
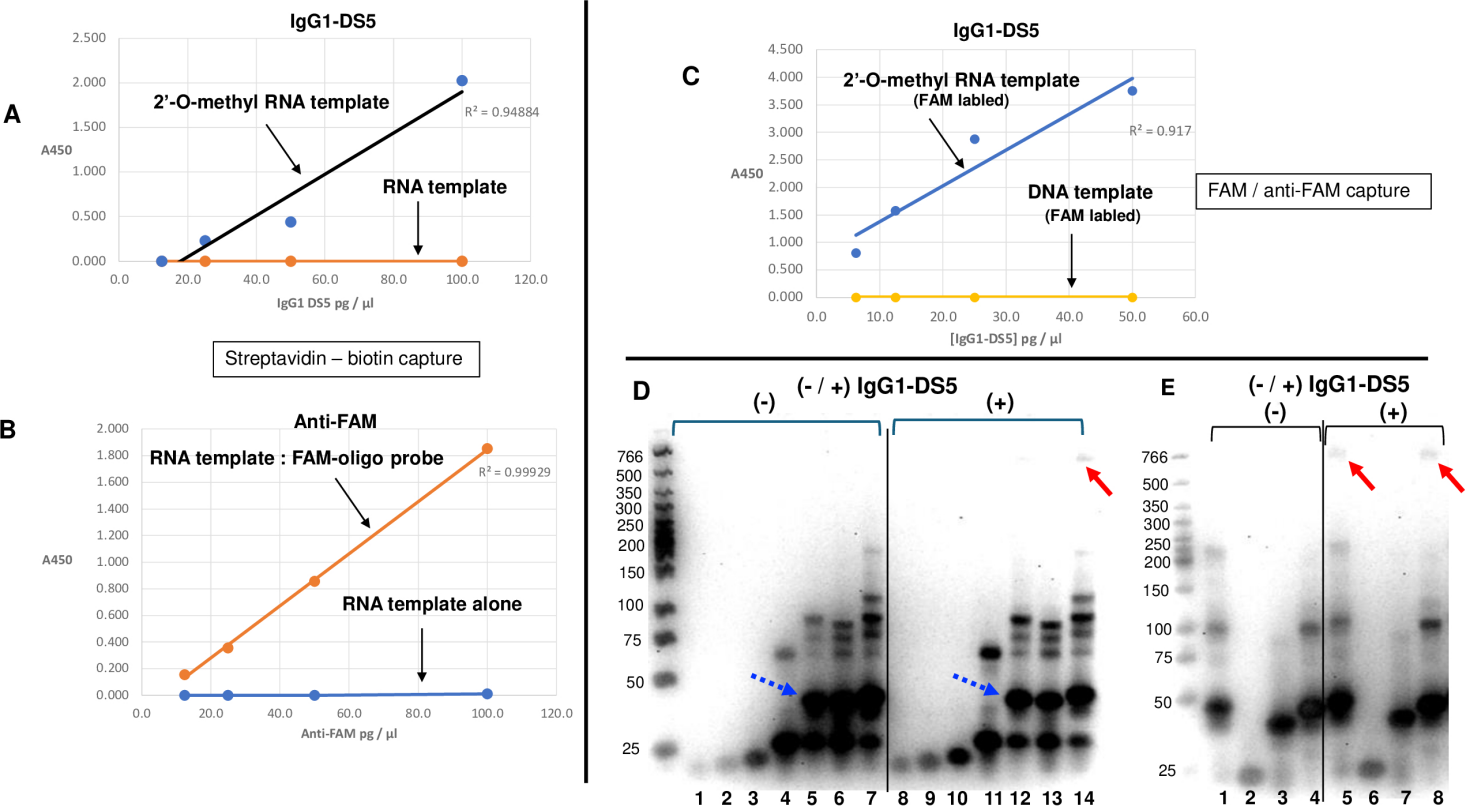
Strand specificity of IgG1-DS5 and gel-shift testing. **(A)** Linear range of ELISA assay for BCN + azide-haplomers on solid-phase 2′-O-methyl RNA vs. RNA templates. **(B)** Response curve for the same plated RNA target template with complementary FAM-labeled oligonucleotides probed with anti-FAM antibody [for both **(A)** and **(B)**, solid-phase capture was via biotinylated templates and streptavidin]. **(C)** ELISA assay for BCN + azide-haplomers on solid-phase 2′-O-methyl RNA vs. DNA templates (via anti-FAM antibody capture of FAM-labeled templates). **(D)** Gel-shift assay for 2′-O-methyl RNA template with single BCN, azide-haplomers, and both. Lanes 1–7: no antibody; lanes 8–14, with IgG1-DS5. Lanes 1, 8=BCN-haplomer alone; lanes 2, 9=azide-haplomer alone; lanes 3, 10=BCN + azide-haplomer; lanes 4, 11=2′-O-methyl RNA template alone; lanes 5, 12=template + BCN-haplomer; lanes 6, 13=template + azide-haplomer; lanes 7, 14=template + both haplomers. **(E)** Gel-shift assay for 2′-O-methyl RNA templates vs. DNA template and BCN, azide-haplomers. Lanes 1–4: no antibody; lanes 5–8: with IgG1-DS5 antibody. Lanes 1, 5=biotin-2′-O-Me template + haplomers 1, 2; lanes 2, 6=haplomers 1, 2 alone; lanes 3, 7=DNA template + haplomers 1, 2; lanes 4, 8=unmodified 2′-O-Me template + haplomers 1, 2. Dashed blue arrows show haplomer:template bands; bold red arrows indicate positions of antibody-shifted bands. Correlation coefficients for linear-range ELISA responses shown as *r*^2^ values. Molecular weights of size marker bands (leftmost lanes) as shown.

By intentional design, all strands in the template:haplomer complex recognized by IgG1-DS5 were composed of 2′-O-methyl RNA. It was thus of interest to determine if the recognition of the template:haplomer complex by IgG1-DS5 was influenced by the specific nucleic acid backbone, as well as the base sequences. No IgG1-DS5 recognition was detected in ELISAs when the biotinylated template 2′-O-methyl RNA strand was replaced with biotinylated RNA itself (**Figure 2A**). In this case, owing to the potential liability of the template RNA strand, FAM-labeled DNA probe oligonucleotides were used to show that a strong signal from anti-FAM antibody was obtained for the FAM-probes hybridized to the plated RNA template, but not with template alone (**Figure 2B**). In a similar fashion, no recognition was seen when haplomers were hybridized to a biotinylated DNA strand with the same haplomer-complementary sequence as for the cognate 2′-O-methyl RNA template (**Figure 2C**).

To investigate both haplomer:template hybridizations and antibody bindings, we used gel-shift assays in 5% non-denaturing TG gels (Methods). Bands with reduced mobility relative to template alone or haplomers alone were seen with combinations of template and single or both haplomers, but a band with retarded mobility in the presence of IgG1-DS5 was only observed for the 2′-O-methyl RNA template with both haplomers (**Figure 2D**). Although haplomer hybridization with a corresponding DNA template was observed, an antibody-induced gel mobility effect was only seen with both haplomers hybridized to a 2′-O-methyl RNA template, where template biotinylation had no effect, as expected (**Figure 2E**).

**Figure 3:**
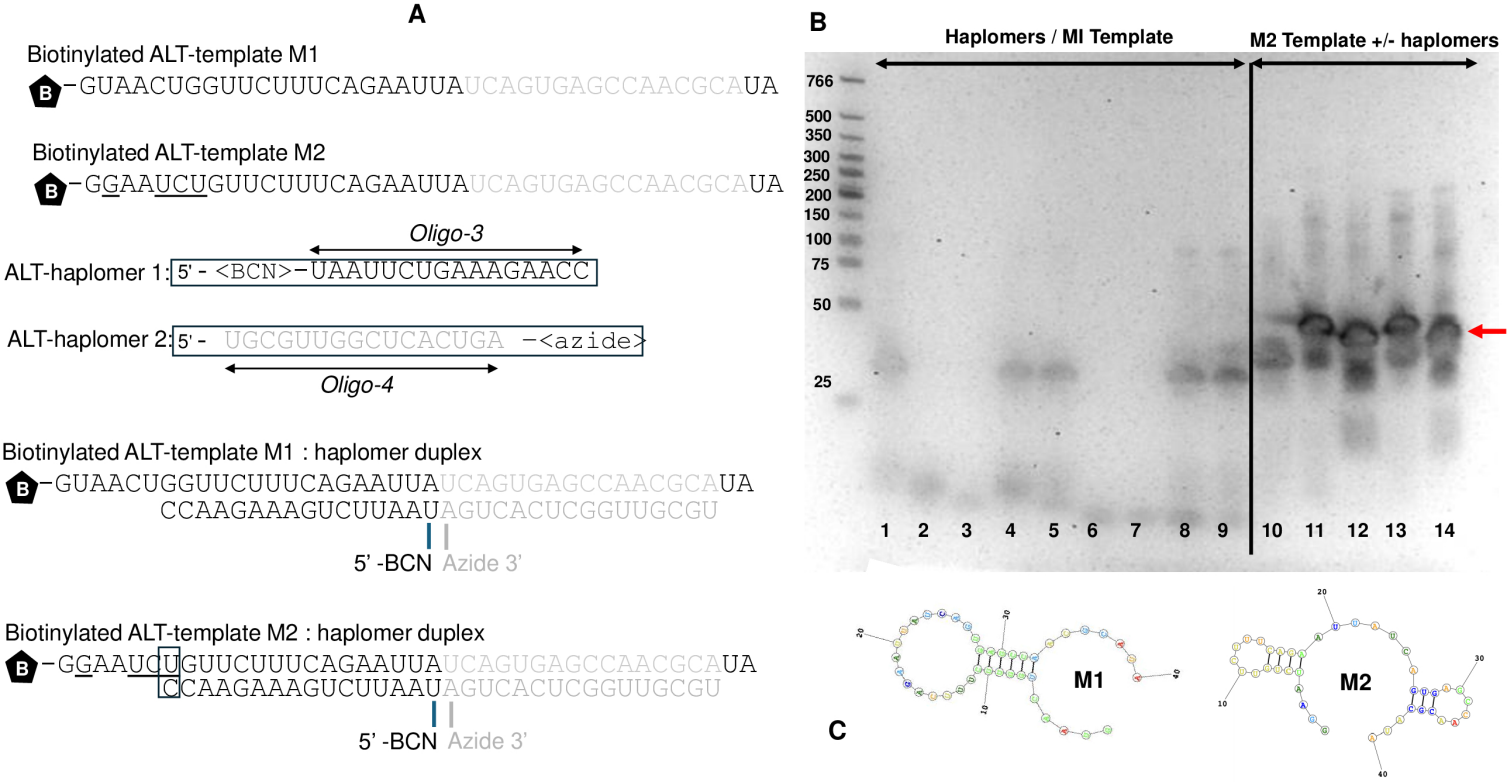
Evaluation of alternative scrambled 2′-O-methyl RNA templates and ALT-BCN, azide-haplomers to be used for IgG1-DS5 recognition specificity determination. Oligo-3, Oligo-4=unmodified sequences corresponding to ALT-BCN and ALT-azide-haplomers, respectively. **(A)** ALT-template M1 and M2 and ALT-haplomer sequences. Base changes in template M2 vs. M1 underlined; boxed base pair shows single-base mismatch between ALT-M2 and ALT-haplomer-1; **(B)** duplex mobility shift tests. Lane 1=M1 template; lane 2=BCN ALT-haplomer-1; lane 3=azide ALT-haplomer-2; lane 4=M1 + BCN ALT-haplomer-1; lane 5=M1 + azide ALT-haplomer-2; lane 6=oligo-3; lane 7=oligo-4; lane 8=M1 + oligo-3; lane 9=M1 + oligo-4; lane 10=M2 template; lane 11=M2 + BCN ALT-haplomer-1; lane 12=M2 + azide ALT-haplomer-2; lane 13=M2 + oligo-3; lane 14=M2 + oligo-4. **(C)** Predicted secondary structures of M1 and M2 templates. Molecular weights of size marker bands (leftmost lane) as shown. Bands with a mobility shift after template:haplomer hybridization shown with a red arrow.

**Figure 4:**
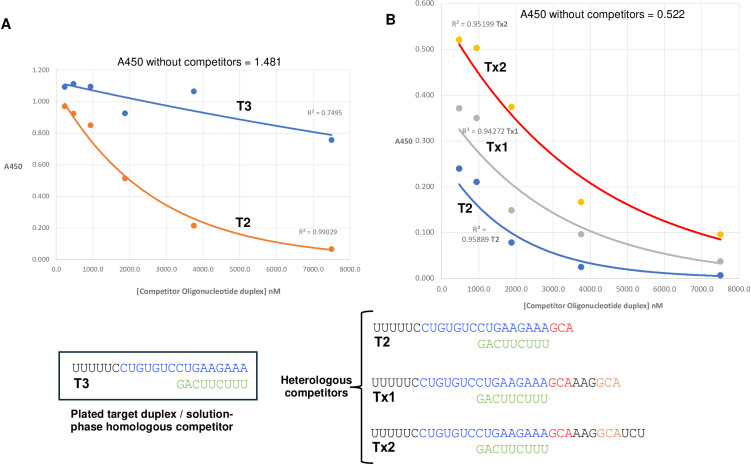
Solution-phase IgG1-DS5 ELISA competition tests with various 2′-O-methyl RNA duplexes toward plated target duplex T3. **(A)** Exponential decay curves for competition with T3 duplex (homologous competitor) vs. T2 duplex. **(B)** Competition decay curves for duplexes with 3-base (T2), 6-base (Tx1), and 9-base (Tx3) 3′-terminal extensions to the top template strand of the T3 duplex. The maximal A450 ELISA values (absence of any competitors) are shown at the top of each plot.

The sequence specificity of IgG1-DS5 recognition was then tested with a scrambled 2′-O-methyl RNA template (M1) and corresponding complementary “ALT” BCN- and azide-haplomers (**Figure 3A**). These were accompanied by oligonucleotides with the same sequences as the ALT-haplomers, but without the BCN or azide modifications (oligo-3 and oligo-4, respectively). Although no recognition of the M1/ALT-haplomers was observed, hybridization between them appeared to be impeded by internal M1 secondary structure, despite initial 80°C preheating of template/haplomer combinations (**Figure 3B**). A subsequent minor sequence alteration predicted to reduce secondary structure (M2 template; **Figure 3C**) did allow hybridization with the same ALT-haplomers despite a single-base mismatch (boxed base pair) as judged by the gel-shift assay shown by strong bands with altered mobilities for the M2 template (**Figure 3B**, lane 10) when hybridized with the ALT-haplomers (**Figure 4B**, lanes 11–14), unlike template M1 (**Figure 3B**, lanes 1–9). Nevertheless, IgG1-DS5 was still unable to recognize the M2:haplomer duplex in ELISA assays (**Supplementary Figure S2**).

### Mapping of binding preferences for IgG1-DS5: short duplex recognition

We subsequently embarked on mapping the fine specificity of IgG1-DS5 target recognition. An initial finding was a surprisingly high level of recognition seen with the template hybridized to the BCN-haplomer alone, while the corresponding template:azide-haplomer showed no measurable binding (**Supplementary Data**, **Supplementary Table S1**; duplexes D1 vs. D2). Moreover, the BCN modification appeared to be superfluous to recognition, as an unmodified oligonucleotide of the same sequence (oligo-1) allowed recognition of comparable degree when hybridized to the template. The unmodified oligonucleotide derived from the azide-haplomer (oligo-2) in similar circumstances was unrecognized (**Supplementary Data**, **Supplementary Table S1**; duplexes D3 vs. D4). Replacing both the BCN- and azide-haplomers with oligo-1 and oligo-2 showed detectable activity, but markedly less than for the template hybridized with oligo-1 alone (**Supplementary Data**, **Supplementary Table S1**; duplexes D3 vs. D5). A template duplex with contiguous 2′-O-methyl RNA oligonucleotides across the original haplomer junction site showed very little or reduced activity (**Supplementary Data**, **Supplementary Table S1**; duplexes D6 and D7).

The apparent preponderance of IgG1-DS5 recognition toward the “left” side of the original target (toward the 5′-end of the template strand) prompted us to investigate the recognition of various truncated unmodified 2′-O-methyl RNA duplexes whose sequences were subsections of the original longer template:haplomer complex (**Supplementary Data**, **Supplementary Table S2**). The simplest duplexes showing apparent recognition completely comparable to the original target were 25- and 22-mers, duplexed with a 9-mer sequence with a 5′-single-stranded region (**Supplementary Table S2**, duplexes T2 and T3). Removing the single-stranded region ablated recognition (**Supplementary Table S2**, duplex T4), as did certain sequence modifications (**Supplementary Table S2**, duplexes T5 and T6). A single base change in the 5′-region of the active duplex T3 (6 bases from the duplex region) was compatible with good recognition (duplex T7), but an additional C→G base change at the boundary of the 9-mer duplex region was completely incompatible with antibody binding (duplex T8). Either scrambling the duplex region (duplex T9) or shifting it within the template itself (duplex T10) likewise could not support recognition. Appending a mismatched pyrimidine sequence to the 3′-end of the bottom (template) strand 9-mer abolished antibody binding (duplex T11), and even extending the 9-mer 3′-end with a fully complementary sequence to the template strand was very poorly compatible with binding activity (duplex T12).

### Competition assays for optimal duplex identification

To further assess the optimal duplex structure recognized by IgG1-DS5, we used competition assays where biotinylated preassembled duplex T3 (**Supplementary Data**, **Supplementary Table S2**) was rendered solid phase by binding to wells of a streptavidin plate and tested for IgG1-DS5 binding, where the antibody was subjected to solution-phase preincubations with dilution series of tested duplex competitors. It was consistently found that duplex T2 (equivalent to T3 but with a 3-base GCA on the top template strand) significantly outperformed homologous duplex T3 itself as a competitor (**Figure 4A**; data best fitted as exponential decay curves with increasing competitor concentrations). Furthermore, 6- and 9-base extensions (duplexes Tx1 and Tx2, respectively) of the T2 3′-GCA sequence (matching the original longer template strand) reduced competitive performance as a function of length (**Figure 4B**). We concluded that the optimal duplex for further studies corresponded to T2 (**Supplementary Data**, **Supplementary Table S2**), with a 25-mer top template strand and a 9-mer complement. Additional competition tests performed in the same manner showed that the recognition of duplex T2 was unaffected by 5′-biotinylation (**Supplementary Figure S2**).

### Sequences of IgG1-DS5 vs. IgG1-DS3 and IgG1-DS5 binding affinity

The protein sequences of IgG1-DS5 and IgG1-DS3 were derived from the coding sequences of the original scFv clones during the original selection process (Methods) and are shown in **Supplementary Data S3**. The CDR regions for IgG1-DS5 and IgG1-DS3 were generally similar (**Table 2**) as might be expected for antibodies selected against a common target. Even so, a notable exception exists in the case of their respective V_H_ CDR3 regions, where the CDR3 of IgG1-DS3 is both significantly divergent and 5 residues longer than the equivalent region for IgG1-DS5 (**Supplementary Table S3****;****Table 2**).

**Table 2:** Comparisons between the CDRs for IgG1-DS5 and IgG1-DS3 (yellow highlight shows matches).

Antibody chain	CDR1	CDR2	CDR3
DS5 V_H_ DS3 V_H_	IDSAG SNSAA	LGRTYYTSKWNNDYAVS LGRTYYRSKWYNDYAVS	DRMVRGVIILDY GRPVRRFGEPRGLYFDY
DS5 V_L_ DS3 V_L_	SGRRSNIGKNSVS SGSSSNIGSSSVS	YDNNKRPS YGNDNRPS	GTWDSSLSAYV ATWDHSLSSVV

**Figure 5:**
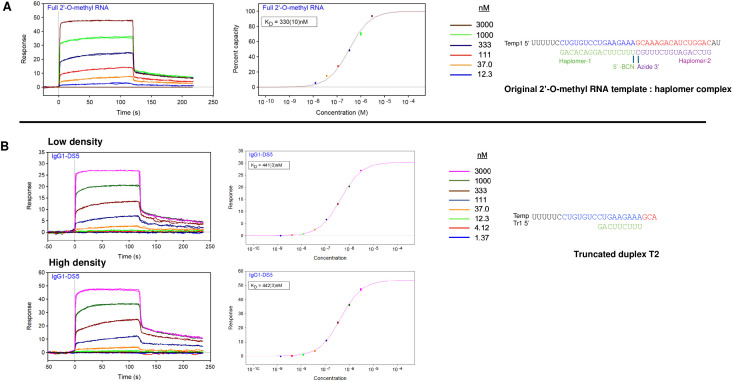
SPR analysis of IgG1-DS5 binding to complete template:haplomer complex and T2 truncated duplex. SPR sensorgrams and steady-state plots for **(A)** original 2′-O-methyl RNA template:haplomer complex and **(B)** truncated duplex T2 are shown. The graphs on the left are the SPR sensorgrams for duplicate injections of six **(A)** or eight **(B)** analyte concentrations ranging between 3,000 and 12.3 nM against captured IgG1-DS5. [In the case of the T2 duplex analyte **(B)**, two captured IgG1-DS5 surfaces were used (low and high density)]. The binding data were collected using a 120 (s) association phase and a 180 (s) dissociation phase. The graphs on the right are the response vs. concentration plots for the analytes binding to IgG1-DS5. For each analyte concentration, the response represents the average binding response collected from 110 to 115 s. The steady-state data were globally fitted to a single-site binding model to determine the equilibrium binding constants (*K*_D_) for each respective analyte.

The key antibody IgG1-DS5 was subjected to SPR analysis in order to define its target binding affinity (Methods). Initially, we used the original template:haplomer complex (**Figure 1**) as the analyte for testing antibody recognition. Data obtained showed complex multiple dissociation rates, diminishing the accuracy of the estimated ***K***_D_ value (~330 nM, **Figure 5A**). We then reasoned that a simpler duplex target might result in an accordingly simplified binding pattern, and SPR tests were repeated with truncated duplex T2 (**Supplementary Data**, **Supplementary Table S2**) lacking a 5′-biotin modification. Nevertheless, the complex dissociation kinetics were still present, with a somewhat reduced estimated *K*_D_ value (~440 nM), independent of antibody ligand surface density (**Figure 5B**). Notable features of the IgG1-DS5 SPR determinations for both the full complex and the short duplex were *R*_max_ values of close to 50% for those expected for a bivalent antibody (Methods).

### Applications for cell surface marker recognition

Though the functional binding states of IgG1-DS5 to either the original or truncated targets were only of mid-range affinities (SPR measurements), its potential practical utility emerged from the experimentally determined (though unexpected) patterns of its binding preferences. Binding of specific short oligonucleotide duplexes is routinely observed, but not toward their component single strands. This unique property of IgG1-DS5 affords a novel opportunity for the development of a proximity-based recognition assay, where each oligonucleotide strand is appended as a tag to molecules binding targets of interest. If two molecules tagged with each oligonucleotide bind to targets arrayed in sufficient molecular proximity, the two strands may anneal to form a duplex, which is then recognizable by IgG1-DS5.

In order to implement this strategy, it is essential that each 2′-O-methyl RNA single-stranded oligonucleotide can be linked to another molecule without ablating the ability of recognizable duplexes to form. Information available showed that duplex recognition was compatible with 5′-biotinylation of the top template strand (duplexes T2, T3, **Supplementary Table S2**), but modifications of the 3′-complementary 9-mer were poorly tolerated if at all (**Supplementary Table S2**), including biotinylation. To test if 5′-modifications of the 9-mer were suitable, we reasoned that an intervening extended PEG linker might help alleviate any interfering steric effects, so a 9-mer 2′-O-methyl RNA with a (PEG)6 linker and a terminal biotin (oligo S9-co) was used. An accompanying top template strand, as in duplex T2 (**Supplementary Table S2**), was also generated with an equivalent biotin-extended linker (S-Tr1). Duplexes of both pairs with one biotinylated strand (biotinylated S-Tr1:non-biotinylated 9-co; non-biotinylated Tr1 template:biotinylated S9-co) were then tested for IgG1-DS5 recognition. It was found that either duplex arrangement was compatible with strong IgG1-DS5 binding in ELISA assays, while both biotinylated single strands were completely unrecognized (**Supplementary Figure S4**). This showed that the single-stranded oligonucleotides could be successfully appended to carrier molecules via their 5′-ends while preserving the recognition of duplexes formed from their hybridizations.

### Use of NAV as an adaptor for oligonucleotides and duplex formation

**Figure 6:**
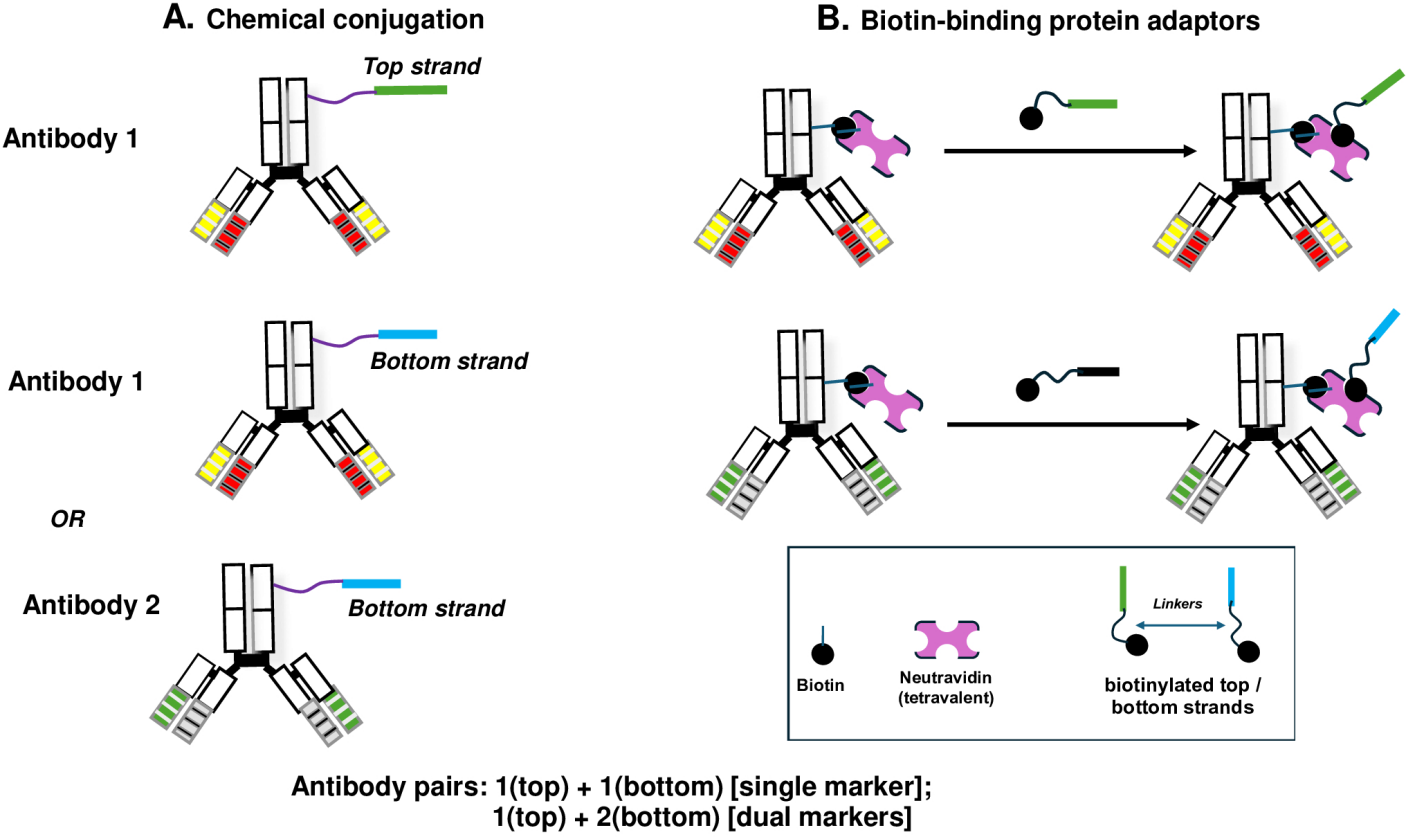
Strategies for appending IgG1-DS5 target oligonucleotides to antibodies against cell surface markers. **(A)** Chemical conjugation, where each strand can be appended to the same antibody against a surface marker, or with a second antibody against a distinct marker. **(B)** Use of a biotin-binding protein, shown as neutravidin (NAV), acting as a tetravalent adaptor between biotinylated antibodies and biotinylated oligonucleotides.

Antibodies have enabled many molecular proximity studies. Where oligonucleotides must be carried by antibodies for this purpose, chemical conjugation can be used to link them together (20). As a simple alternative for enabling oligonucleotide–protein linking, we have manipulated the tetravalency of biotin-binding proteins such as streptavidin, avidin, or NAV (21). For using NAV as an adaptor, biotinylated duplexes or single strands can be linked to biotinylated antibodies (widely commercially available) as depicted in **Figure 6**. Regardless of the method for attaching oligonucleotides to antibodies, formation of a duplex recognized by IgG1-DS5 can be in principle be instigated by annealing of conjugated strands forced into proximity by binding of carrier antibodies to their cognate targets. This can be mediated by separate single strands attached to the same antibody (homologous pairs), or to distinct antibodies (heterologous pairs), provided antibody binding juxtaposes complementary strands into sufficiently close proximity for hybridization to occur (**Figure 6**).

### Metabolic labeling for testing IgG1-DS5 target duplex cell surface recognition

**Figure 7:**
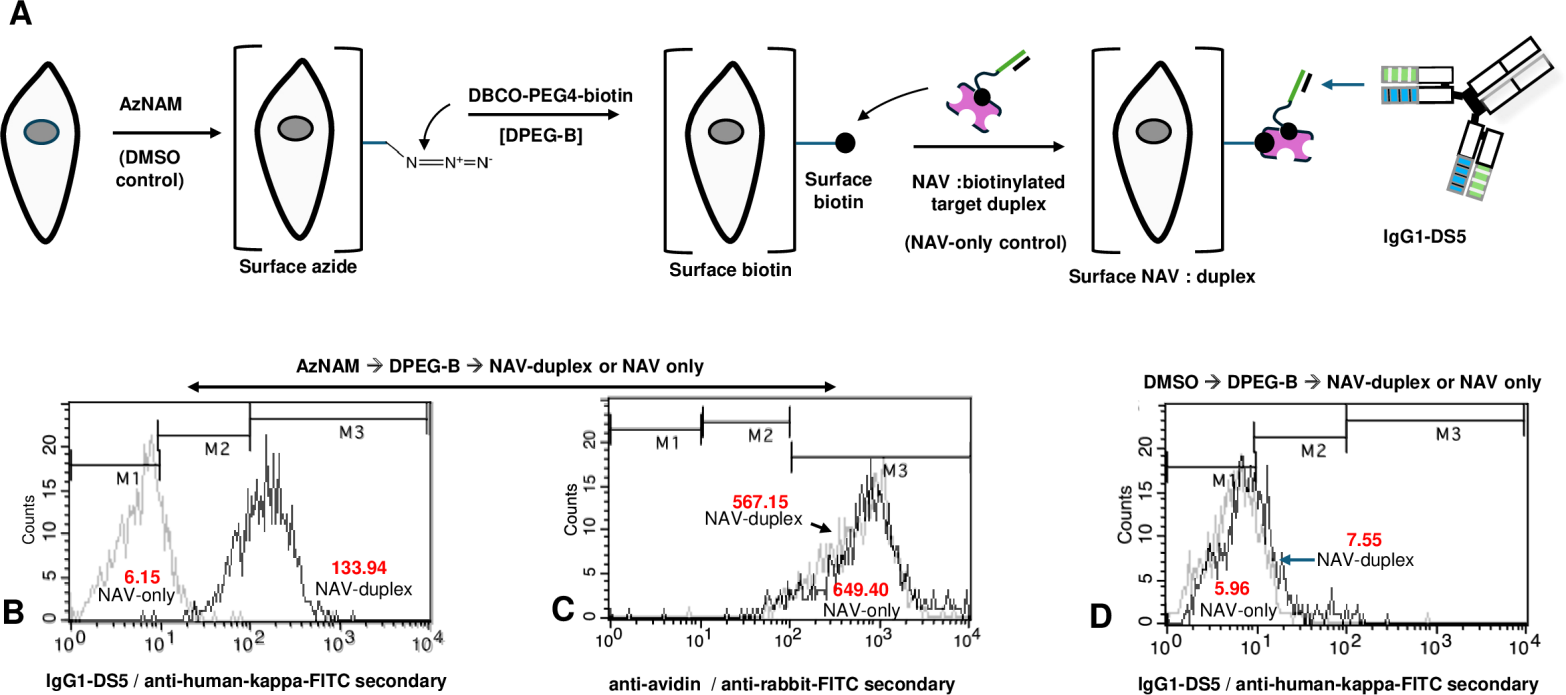
Recognition of target 2′-O-methyl RNA duplex on SKBr3 cell surfaces by IgG1-DS5. **(A)** Rendering of cells with surface biotins via metabolic labeling, for surface localization of neutravidin (NAV) bearing biotinylated IgG1-DS5 target duplex. Symbols for biotin and target 2′-O-methyl RNA strands as for **Figure 6**. Cells are initially treated with AzNAM (peracetylated N-azidoacetylmannosamine) solubilized in DMSO or DMSO solvent control alone (Methods). Uptake of AzNAM (24 h) enables metabolic surface positioning of terminal azide moieties, which are then reacted with the compound dibenzocyclooctyne-[DBCO]-PEG4-biotin (DPEG-B). Surface biotins can then be bound by NAV alone or NAV bearing subsaturating amounts of biotinylated 2′-O-methyl RNA target duplexes recognizable by IgG1-DS5. **(B)** Flow analysis of AzNAM/DPEG-B/NAV alone or NAV-duplex-treated cells with IgG1-DS5 (with secondary antibody goat anti-human kappa chain-FITC conjugate). **(C)** Flow analysis of treated cells as in **(B)** with anti-avidin (with goat anti-rabbit-FITC conjugate). **(D)** Flow analysis with IgG1-DS5 as in **(B)**, but with initial DMSO controls instead of AzNAM treatments. Figures in red text show geometric mean fluorescence values.

The use of a biotin-based system for placement of oligonucleotide targets is also compatible with metabolic labeling systems. We initially used this approach to demonstrate that IgG1-DS5 could recognize a defined target duplex on a cell surface. For this purpose, SKBr3 cells were treated with peracetylated N-azidoacetylmannosamine (AzNAM, Sigma, Saint Louis, MO, USA), which allows the metabolic positioning of azide moieties as terminal surface glycan sialate residues (Methods). These surface azides can subsequently react with bifunctional compounds containing click-reactive moieties, as with DBCO (dibenzocyclooctyne)-PEG4-biotin (BroadPharm, San Diego, CA, USA). Such treatments in effect coat cell surfaces with the desired biotin functional group, in turn permitting binding of NAV alone, or NAV bearing subsaturating amounts of biotinylated target 2′-O-methyl RNA duplexes (**Figure 7A**). It was found by flow analyses (**Figure 7B**) that NAV-duplexes were strongly recognized by IgG1-DS5 on biotin-labeled cells, with no such signals arising from NAV alone [highly significant by *t*-test analysis (Methods); *p*<0.0001]. In contrast, probing both sets of cells with anti-avidin antibody (which binds to NAV) gave strong and superimposable signals, proving that NAV was present in both NAV-only and NAV-duplex sets (**Figure 7C**). Cells initially treated with the solvent DMSO alone instead of AzNAM (solubilized in DMSO) do not display surface azide or biotins, and accordingly, no IgG1-DS5 binding was observable (**Figure 7D**).

### Antibody-mediated target duplex and surface-annealed single strand recognition

**Figure 8:**
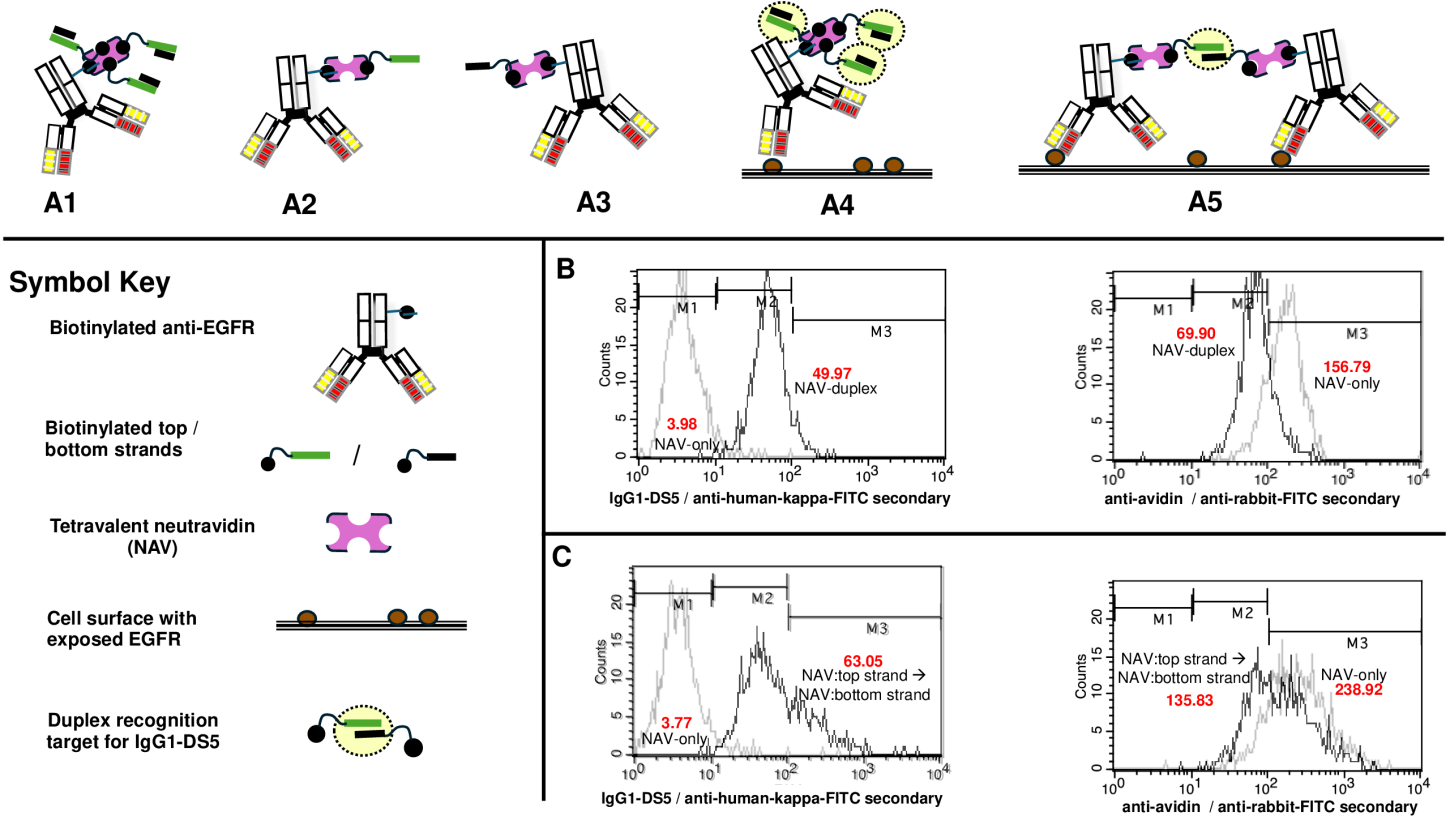
Recognition by IgG1-DS5 of either preformed target 2′-O-methyl RNA duplexes or surface-annealed single strands on SiHa cells, both via monoclonal anti-EGFR antibody (R&D Systems, Minneapolis, MN, USA). **(A1–3)** Biotinylated anti-EGFR complexed with duplex target **(A1)**; target top strand **(A2)** and target bottom strand **(A3)**. For **(A2)** and **(A3)**, diagrams shown with subsaturating oligonucleotide binding for simplicity. **(A4)** Biotinylated anti-EGFR complexed with duplex target on the cell surface via EGFR binding. (A5) Formation of a duplex target by annealing of single strands on a cell surface via proximal anti-EGFR binding. **(B)** Flow analyses for NAV-duplexes vs. NAV-only controls with anti-IgG1-DS5 or anti-avidin. Left panel: anti-IgG1-DS5 probe/goat anti-human kappa chain FITC secondary; right panel: anti-avidin probe/goat anti-rabbit FITC secondary. **(C)** Formation of duplex targets via separate sequential bindings of anti-EGFR bearing single strands via NAV adaptors. Left panel: anti-IgG1-DS5 probe/goat anti-human kappa chain FITC secondary; right panel: anti-avidin probe/goat anti-rabbit FITC secondary. Figures in red text show geometric mean fluorescence values.

We then proceeded to test for cell surface formation of duplexes recognizable by IgG1-DS5. For this aim, we used a single biotinylated antibody capable of binding to the well-characterized surface receptor EGFR (R&D Systems, Minneapolis, MN, USA), shown to be expressed on SKBr3 and SiHa cells (**Supplementary Figure S5**). This biotin-antibody was initially treated with NAV bearing preformed biotinylated target duplex T2 (**Supplementary Data**, **Supplementary Table S2**) or its component 5′-biotinylated single strands (**Figure 7A1-3**). As with the above metabolic labeling system, antibody-mediated placement of surface duplex was successful (*p*<0.0001; Methods), with no binding of IgG1-DS5 to NAV alone but strong anti-avidin signals in each case (**Figure 8B**).

**Figure 9:**
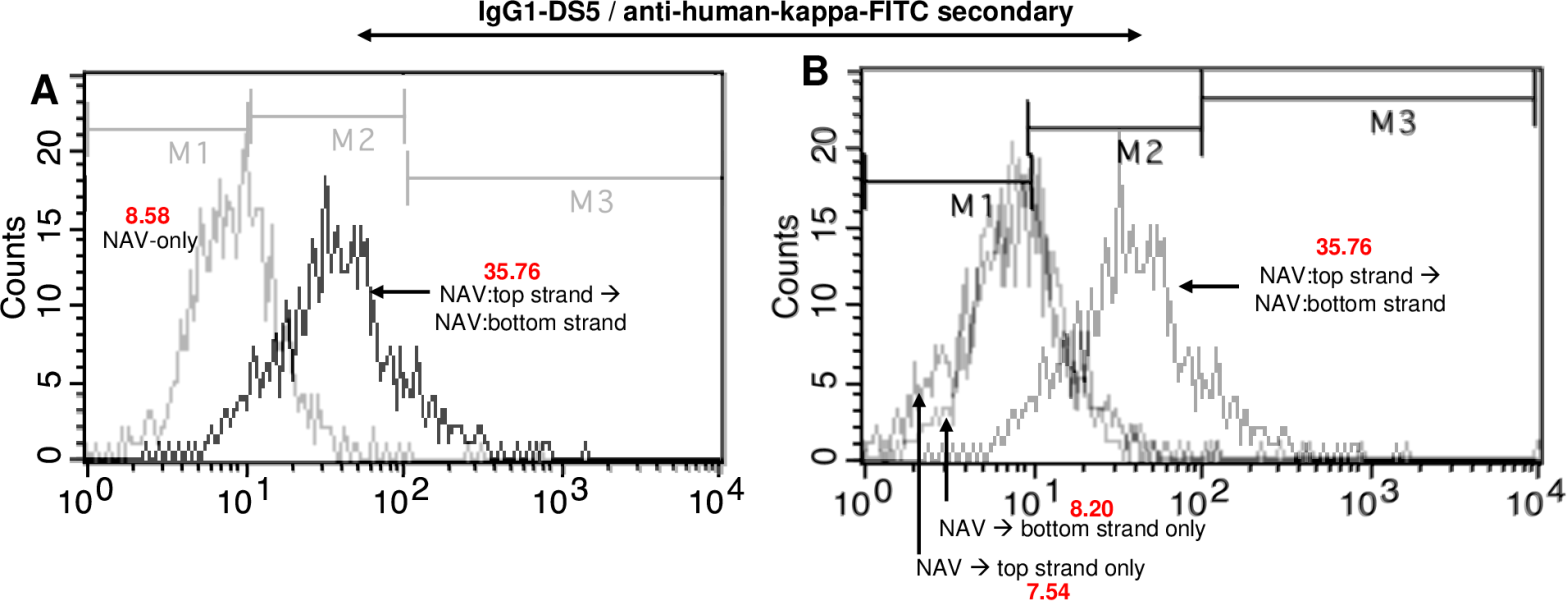
Recognition by IgG1-DS5 of surface-annealed single strands on SKBr3 cells via anti-EGFR antibody (biotinylated goat polyclonal, R&D Systems, Minneapolis, MN, USA), with NAV-only and single-strand-only controls. **(A)** Flow analyses for duplex targets via separate sequential bindings of anti-EGFR bearing single top and bottom strands via NAV adaptors vs. NAV-only controls, with IgG1-DS5 probe/goat anti-human kappa chain FITC secondary. **(B)** Flow analyses for duplex targets via separate sequential bindings of anti-EGFR bearing single top and bottom strands via NAV adaptors vs. corresponding tests with top and bottom single strands only, with IgG1-DS5 probe/goat anti-human kappa chain FITC secondary. Figures in red text show geometric mean fluorescence values.

This was followed by antibodies tagged via NAV with single strands to assess the formation of surface duplexes (**Figure 8A5**). To avoid the possibility of strand annealing occurring in solution prior to binding of the anti-EGFR antibody to its surface target, we used sequential additions of first top-strand antibody, a wash step, and then the second-strand antibody (Methods). Highly significant signals were observed in the sequential binding set, but not where both steps involved NAV alone (**Figure 8C**; *p*<0.0001; Methods). Both procedures allowed readily detectable cell surface NAV, as assessed with anti-avidin (**Figure 8C**). When the NAV bearing only single strands rather than sequential strand additions was used, no signals above background were observed (**Figures 9A,B**).

## Discussion

The data presented demonstrate the unique specificity of a novel antibody that binds to short 2′-O-methyl RNA duplexes while showing no recognition toward their single-stranded components. The isolated antibody was selected to facilitate studies of epitopes produced with nucleic acid duplex formation that occurs when click reactions are allowed by proximity of binding to nucleic acid templates. While we anticipated that selected antibodies would include reactivity toward click modifications, a markedly unexpected binding specificity was obtained that was not dependent on the original click modifications within the starting complex. The resulting antibody IgG1-DS5 binds to short 2′-O-methyl RNA duplexes while showing no recognition toward their single-stranded components. Moreover, this antibody has both nucleic acid backbone specificity and a high requirement for sequence preservation for significant recognition to be maintained. While anti-DNA and anti-RNA antibodies have been reported, some with significance in certain pathologies (22), to our knowledge, no antibody specifically binding to 2′-O-methyl RNA has ever been described nor toward any nucleic acid with the short duplex-specific characteristics of IgG1-DS5. Therefore, there is some inherent interest in the binding properties of this antibody in its own right. Of particular import is IgG1-DS5 recognition of a fully bioorthogonal structure that would not be anticipated to occur in mammalian cells, making this antibody uniquely useful for both diagnostic and potential therapeutic uses. The companion antibody IgG1-DS3 also bound the original 2′-O-methyl RNA:haplomer target but showed reduced selectivity in binding to template alone (**Figure 2B**). Although such template-only ELISA responses with IgG1-DS3 consistently indicated lower affinities than those observed toward the full complex, the specific binding pattern of IgG1-DS5 was favored for the original project aims. It is inferred that the differences in the respective CDR sequences between these two antibodies (particularly the marked variation in their V_H_ CDR3 sequences) account for their distinct binding specificities, but a full understanding of this will require future structural studies.

The binding of IgG1-DS5 to an unmodified 2′-O-methyl RNA duplex was especially surprising given that during the selection process from scFv phage libraries, counterselections were applied designed to remove binders toward single-stranded template and haplomers (Methods). From SPR analysis, the mechanism of binding showed a complex pattern even with the simplified duplex target, with multiple apparent dissociation rates. The molecular significance of this remains to be determined, but it is possibly related to IgG1-DS5 forming interchangeable binding registers of variable affinities with the duplex target. It was also of interest that the *R*_max_ value was repeatedly close to 50% of the predicted level, which would be consistent with univalent rather than typical bivalent antibody binding. It is conceivable that the size of even the minimal duplex acts as a steric constraint that reduces the possibility of simultaneous bivalent binding of two duplex copies by each V_H_:V_L_ antibody chain pair.

Although the measured binding affinity of IgG1-DS5 toward a minimal duplex was not high level (~440 nM), the unique properties of this antibody nevertheless provided an opportunity to test whether it could be exploited as a novel reagent in molecular proximity assays. For such applications, it is necessary to append oligonucleotides to third-party molecules binding to individual markers of interest, which has involved antibodies and chemical conjugation in the case of the existing PLA. As an alternative to the chemical linkage strategy, we developed a facile alternative using biotinylation of both a designated antibody and the individual single strands composing the duplex recognized by IgG1-DS5, combined with a tetravalent biotin-binding protein as an adaptor. Almost all antibodies directed against cellular surface targets of interest are commercially available in biotinylated form, and if necessary, custom biotinylation kits can be applied. Terminal biotinylation of synthetic oligonucleotides is routinely offered by numerous manufacturers. With respect to the necessary biotin-binding protein with tetravalency, either readily available streptavidin or avidin is an option. The commercially available NAV version of avidin has the theoretical advantage for cell studies of the absence of a putative integrin-binding sequence (Thermo Fisher Scientific, Waltham, MA, USA) and (in common with streptavidin) a near-neutral p***I*** value. The success of cellular proximity assays *in vitro* (**Figures 7**, **8**) suggests that with its present affinity level, IgG1-DS5 is a useful reagent in this regard.

As well as chemical conjugation via an oligonucleotide modified with an amine- or sulfhydryl-reactive moiety (20), alternative site-specific conjugation methods have been developed that are applicable to antibody–oligonucleotide conjugate formation. These include approaches mediated by sortase (23), SNAP-tag, and HaloTag (24). Site-specific HUH endonucleases have also attracted interest for generating protein–oligonucleotide conjugates for varied applications (25). Although native HUH enzymes are DNA-specific, it has been possible by directed engineering to convert the strand specificity toward RNA (26), so it may be equally feasible to generate a modified HUH activity toward a nuclease-resistant nucleic acid. The latter would be advantageous for a process involving labeled antibodies (as for IgG1-DS5) with potential *in vivo* applications. While these approaches may be useful in the future, for the present proof-of-concept work, we opted for the use of a tetravalent biotin-binding adaptor (NAV), due to its compatibility with readily available biotinylated antibody reagents and to avoid the need for expression of tagged fusion proteins.

While NAV-based linkage of antibodies (or other biotinylated molecules) is easily applied, it is important to note certain caveats. While several protocols are possible, we have found that the best way to generate the final antibody:NAV:oligonucleotide is to initially bind the NAV to the biotinylated antibody and then add a superexcess of biotinylated target duplex or single strands to saturate available free biotin-binding sites. For the initial NAV-binding step, calculating the theoretical molar ratios is obviously dependent on the biotinylation ratio of the antibody, which will vary from batch to batch (and manufacturer), but an approximate guideline was provided as 10 biotins per antibody molecule from R&D Systems, Minneapolis, MN, USA. A preliminary empirical test for optimal ratios for biotin-antibody:NAV binding is useful, keeping in mind that too high an excess of antibody may encourage cross-linking, while excess NAV may tend toward saturation of available biotin-binding sites. The addition of a final superexcess of either biotinylated duplex or single strands is then applied to ensure saturation of all remaining biotin-binding sites. Once defined for a specific biotin-antibody, the same conditions for complex formation can be routinely applied.

Though binding of IgG1-DS5 to duplex targets was easily demonstrable with ELISA assays, it could not be assumed that this would still apply on cell surfaces, especially given the unprecedented recognition specificity of this antibody. To test this initially, we put the NAV–oligonucleotide–biotin system to good use via metabolic labeling, for positioning first cell surface azides and subsequently [via a secondary click reaction (Methods)] surface biotin residues (**Figure 7A**). This strategy enabled NAV alone or NAV carrying subsaturating amounts of biotinylated target duplex to bind to cell surfaces bearing biotins. We then showed that NAV carrying target duplexes gave strong signals from biotinylated cells in the flow analyses with the IgG1-DS5 probe, whereas no signals above background were seen with NAV alone (**Figure 7B**). To confirm that NAV was nonetheless bound in both circumstances, we then showed that anti-avidin elicited signals from both NAV-duplex and NAV-alone treatments (**Figure 7C**). In contrast, when the DMSO solvent was applied to cells rather than AzNAM, no IgG1-DS5 signal was seen as expected, since no surface biotins could be produced (**Figure 7D**).

For the enablement of cellular proximity-based assays with IgG1-DS5, it is essential that annealing can occur between separate strands carried by antibodies (or other molecules) binding to cell surface markers. As proof of principle for this aim, we used biotinylated anti-EGFR antibodies combined with NAV carrying either duplexes (as a control for IgG1-DS5 activity, as cell surface duplex binding was initially confirmed; **Figure 7**) or either of the two single strands comprising the duplex. Initially, we showed by ELISA that with suitable linkers, 5′-biotinylation of either top template or bottom strand could act as a functional anchor to streptavidin to enable the annealing of complementary non-biotinylated strands (**Supplementary Figure S4**). Since either arrangement was equally well recognized by IgG1-DS5, it was thus shown that single strands could be functionally deployed for proximity-based annealing via linkage to a biotin-binding protein. We then used SiHa cells (known to express significant EGFR levels; **Supplementary Figure S5**) to initially demonstrate that cell-bound biotinylated anti-EGFR antibody complexed with NAV-duplexes could successfully elicit strong signals from IgG1-DS5, but only background with NAV-only. Cells bearing either NAV-duplexes or NAV alone (via initial binding of the carrier anti-EGFR antibody), nonetheless, both gave strong signals with anti-avidin, showing that the NAV protein was clearly present in both cases (**Figure 8B**).

We then used complexes of anti-EGFR carrying NAV:oligonucleotides to test for the ability of such strands to anneal on target cell surfaces (via primary carrier antibody binding to surface EGFR), and thus, in turn, form target structures for IgG1-DS5. Here, we used sequential additions of each antibody:NAV:oligonucleotide complexes, involving an initial incubation for the first strand followed by washing and then a second incubation with the bottom strand (Methods). This was done in order to mitigate possible solution-phase annealing if both antibody complexes were added simultaneously. It was found that such sequential strand additions (with a final incubation to allow time for annealing) allowed IgG1-DS5 recognition, but not for the antibody-NAV alone (**Figure 8C**). With another EGFR-positive cell line (SKBr3; **Supplementary Figure S5**), the same single-strand sequential addition also allowed a strong IgG1–51 signal, but again not with anti-EGFR complexed with NAV alone, nor with NAV where only single strands were added (**Figure 9**).

The utility of a single-antibody therapy applied to abnormal tumor-related surface expression is exemplified by trastuzumab for HER2+ breast cancer (27), especially when the trastuzumab is conjugated with highly cytotoxic drugs such as emtansine or deruxtecan (28, 29). Nevertheless, since HER2 cell surface expression *per se* is not restricted to tumor cells, unwanted “bystander” effects can occur, and cardiotoxicity arising from heart HER2 expression is a well-characterized deleterious trastuzumab side effect (30, 31). If a system is devised whereby a therapeutic response is engendered not merely by the presence of a marker in itself, but is intimately coupled with marker surface density, then the desired response can be directed more exclusively toward cells with pathologically high marker densities. Formation of duplexes (recognized by IgG1-DS5) via tagged antibodies that bind surface markers in high density may provide a way to provide such a beneficial focusing on cancer cells while limiting toxicity toward normal tissues.

For the purposes of this initial investigation into the potential utility of IgG1-DS5, we have used a single antibody against a surface marker (EGFR), where the anti-EGFR is tagged with both strands comprising the optimal DS5 duplex, for surface annealing as described above. Adapting such an arrangement to a system with two primary antibodies against different surface markers (each primary antibody bearing either the top or the bottom strand of the target duplex) can be readily undertaken for further development of the IgG1-DS5 functional repertoire.

Future improvements in the performance of IgG1-DS5 itself can be envisaged, including AI-predicted structure-guided sequence tuning of CDRs to enhance binding affinity, which may be of particular importance for future *in vivo* studies. Alternatively, multimeric forms of IgG1-DS5 Fab fragments could be engineered in order to produce an avidity gain for boosting effective binding persistence. While the latter effect may prove useful *in vivo*, the results presented here show that IgG1-DS5 itself is clearly effective at least for *in vitro* assays as a facile alternative to the PLA.

The unique recognition of bioorthogonal surface-annealed duplexes by the antibody IgG1-DS5 has many foreseeable future applications, since there is no limitation in principle for its deployment for *in vivo* purposes. In particular, IgG1-DS5 could be modified with either a fluorescent marker or a radioisotope (for diagnostic/visualization applications) or with a highly cytotoxic drug for cancer therapy, such as deruxtecan, as used with trastuzumab (32, 33). The ability of the oligonucleotide duplex formation system to exploit molecular proximity extends to dual marker pairs, and there are many potential types of primary binding molecules that may be used. Thus, beyond antibodies alone, a variety of receptor–ligand systems could be tagged for single-stranded oligonucleotides, either as homologous or heterologous pairs (as exemplified by folate and folate receptor systems; **Supplementary Figure S6**).

## Data Availability

The data presented in the study are deposited in the GenBank repository, accession numbers PX753276 (scFv-DS3) and PX753276 (scFv-DS5).
