# Supplementary material for: An antibody uniquely binding short 2′-O-methyl RNA oligonucleotide duplexes: formation and recognition of target duplexes on cell surfaces
Source: Front Immunol. 2026 Jan 12;16:1699400. doi: 10.3389/fimmu.2025.1699400 (PMC12833424; doi:10.3389/fimmu.2025.1699400)
Supplement: Supplementary file 1 [file DataSheet1.zip › Supplementary Material.jpg/SupplementaryTables&Data.docx]

Table S1. ELISA testing of original duplex and variants. No signals obtained from any biotinylated templates alone.

| Code | Duplex | Status | ELISA response * (% full complex) |
| --- | --- | --- | --- |
| D0 |  | Template duplex used for scFv library selection; both haplomers 1 (BCN) and 2 (Azide) | 100.0 |
| D1 |  | Template duplex, BCN-haplomer only | 71.5 |
| D2 |  | Template duplex, Azide-haplomer only | 0.0 |
| D3 |  | Template duplex, with complementary strand corresponding to unmodified haplomer 1 (oligo-1) only | 76.1 |
| D4 |  | Template duplex, with complementary strand corresponding to unmodified haplomer 2 (oligo-2) only | 0.0 |
| D5 |  | Template duplex, with complementary strands corresponding to haplomers 1 and 2 without modifications (oligo-1 and oligo-2) | 45.0 |
| D6 |  | Template duplex, with contiguous complementary strand-1 | 0.8 |
| D7 |  | Template duplex, with contiguous complementary strand-2 | 40.0 |

All top-strand templates biotinylated; all strands 2’-O-methyl RNA

* % of IgG1-DS5 ELISA slope curves of specific template / haplomer or oligo duplexes compared with original full complex

Oligo-1 = #1054; Oligo-2 = #1049

Table S2. ELISA testing of truncated duplexes and variants. No signals obtained from any biotinylated templates alone.

| Code | Duplex | Status | ELISA response * (% standard complexes) |
| --- | --- | --- | --- |
| T1 |  | Template truncated from 3’ end of Temp1 (25-mer), Oligo-1 | 100 |
| T2 |  | Template truncated from 3’ end of Temp1 (25-mer), Oligo-1 truncated from 3’-end (9-mer duplex) | 100 |
| T3 |  | Template truncated from 3’ end of Temp1 (22-mer), Oligo-1 truncated from 3’-end (9-mer duplex) | 100 |
| T4 |  | Template truncated from 3’ end of Temp1 with internal deletion CCUGUGUC (14-mer), Oligo-1 truncated from 3’-end (9-mer duplex) | 0 |
| T5 |  | As for T3 except R/Y sequence alterations in 22-mer template single-stranded region | 0 |
| T6 |  | As for T3 except (GU)4 sequence pattern in 23-mer template single-stranded region | 0 |
| T7 |  | As for T3 except (GU)3-C sequence pattern in 22-mer template single-stranded region | 94.5 |
| T8 |  | As for T8 except (GU)3-G sequence pattern in 22-mer template single-stranded region | 0 |
| T9 |  | As for T3 except 9-mer 3’-template region scrambled with complementary oligo to form 9-mer duplex | 0 |
| T10 |  | As for T3 except 9-mer 3’-template region transposed towards 5’-end of template strand, same 9-mer duplex | 0 |
| T11 |  | As for T2 but with mismatched 3’-extension from 9-mer duplex | 0 |
| T12 |  | As for T2 but with complete complementarity from 9-mer duplex | 6.0 |

All top-strand templates biotinylated; all strands 2’-O-methyl RNA

* % of IgG1-DS5 ELISA slope curves of specific template / haplomer or oligo duplexes compared with original full complex (T1-T3) or against T2 as a comparative standard (T4 – T12)

Supplementary data S3: IgG1-DS5 and IgG1-DS3 V_H_ and V_L_ sequences

IgG1-DS5 as original scFv sequence:

N-terminus

QVQLQQSGPGLVKPSQTLSLTCAIFGDSVSIDSAGWNWIRQSPSRGLEWLGRTYYTSKWNNDYAVSVKSRITINPDTSKNQFSLQLNSVTPEDTAVYYCARDRMVRGVIILDYWGKGTLVTVSSGGGGSGGGGSGGGGSQSVVTQPPSVSAAPGQKVTISCSGRRSNIGKNSVSWYQHLPGTAPKLLIYDNNKRPSGIPDRFSGSRSGTSATLGITGLQTGDEADYYCGTWDSSLSAYVFGTGTKVTVLG

IgG1-DS5 V_H_

QVQLQQSGPGLVKPSQTLSLTCAIFGDSVSIDSAGWNWIRQSPSRGLEWLGRTYYTSKWNNDYAVSVKSRITINPDTSKNQFSLQLNSVTPEDTAVYYCARDRMVRGVIILDYWGKGTLVTVS

IgG1-DS5 V_L_

QSVVTQPPSVSAAPGQKVTISCSGRRSNIGKNSVSWYQHLPGTAPKLLIYDNNKRPSGIPDRFSGSRSGTSATLGITGLQTGDEADYYCGTWDSSLSAYVFGTGTKVTVLG

Shaded residues = ser-gly linker; Red residues = heavy chain CDRs, blue = light chain CDRs; in order from N-termini towards C-termini, for CDR1, CDR2, and CDR3.

--- --- --- --- --- --- --- --- --- --- --- --- --- --- --- --- --- --- --- --- --- --- --- --- --- --- --- --- --- --- --- --- --- --- --- --- --- --- --- --- --- --- ---

Supplementary data S3: IgG1-DS5 and IgG1-DS3 V_H_ and V_L_ sequences, continued

IgG1-DS3 as original scFv sequence:

N-terminus

QVQLQQSGPGLVKPSQTLSLTCAISGDSVSSNSAAWNWIRQSPSRGLEWLGRTYYRSKWYNDYAVSVKSRITINPDTSKNQFSLQLNSVTPEDTAVYYCARGRPVRRFGEPRGLYFDYWGKGTLVTVSSGGGGSGGGGSGGGGSQAVLTQPPSASGTPGQRVTISCSGSSSNIGSSSVSWYQQLPGAAPKLLIYGNDNRPSGVPDRFSGSKSGTSATLGITGLQTGDEADYYCATWDHSLSSVVFGKGTKVTVLG

IgG1-DS3 V_H_

QVQLQQSGPGLVKPSQTLSLTCAISGDSVSSNSAAWNWIRQSPSRGLEWLGRTYYRSKWYNDYAVSVKSRITINPDTSKNQFSLQLNSVTPEDTAVYYCARGRPVRRFGEPRGLYFDYWGKGTLVTVS

IgG1-DS3 V_L_

QAVLTQPPSASGTPGQRVTISCSGSSSNIGSSSVSWYQQLPGAAPKLLIYGNDNRPSGVPDRFSGSKSGTSATLGITGLQTGDEADYYCATWDHSLSSVVFGKGTKVTVLG
