# Supplementary figures and images for: An antibody uniquely binding short 2′-O-methyl RNA oligonucleotide duplexes: formation and recognition of target duplexes on cell surfaces
Source: Front Immunol. 2026 Jan 12;16:1699400. doi: 10.3389/fimmu.2025.1699400 (PMC12833424; doi:10.3389/fimmu.2025.1699400)

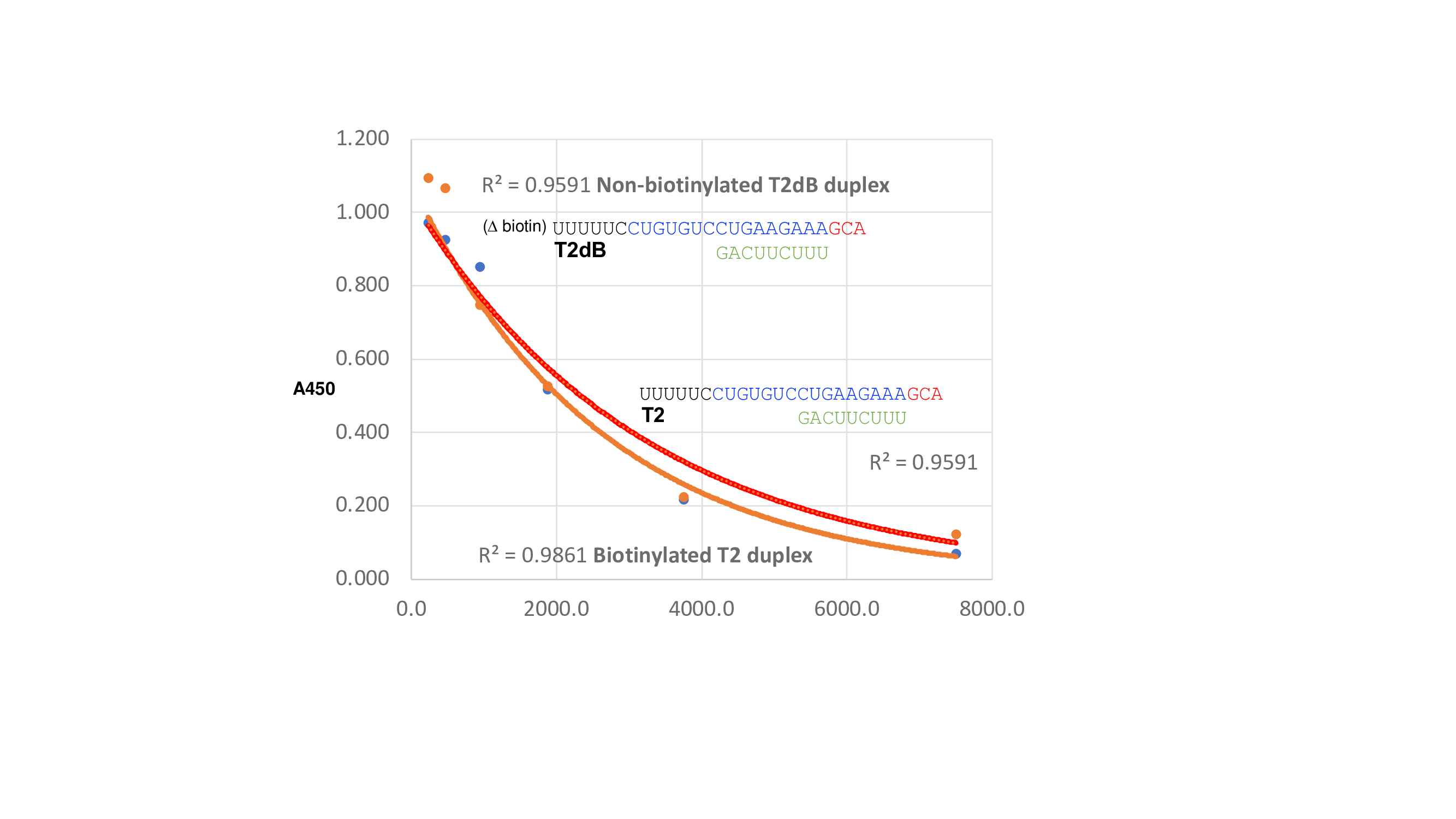

Supplement: Supplementary file 1 [file DataSheet1.zip › Supplementary Material.jpg/Figure S3.jpg]

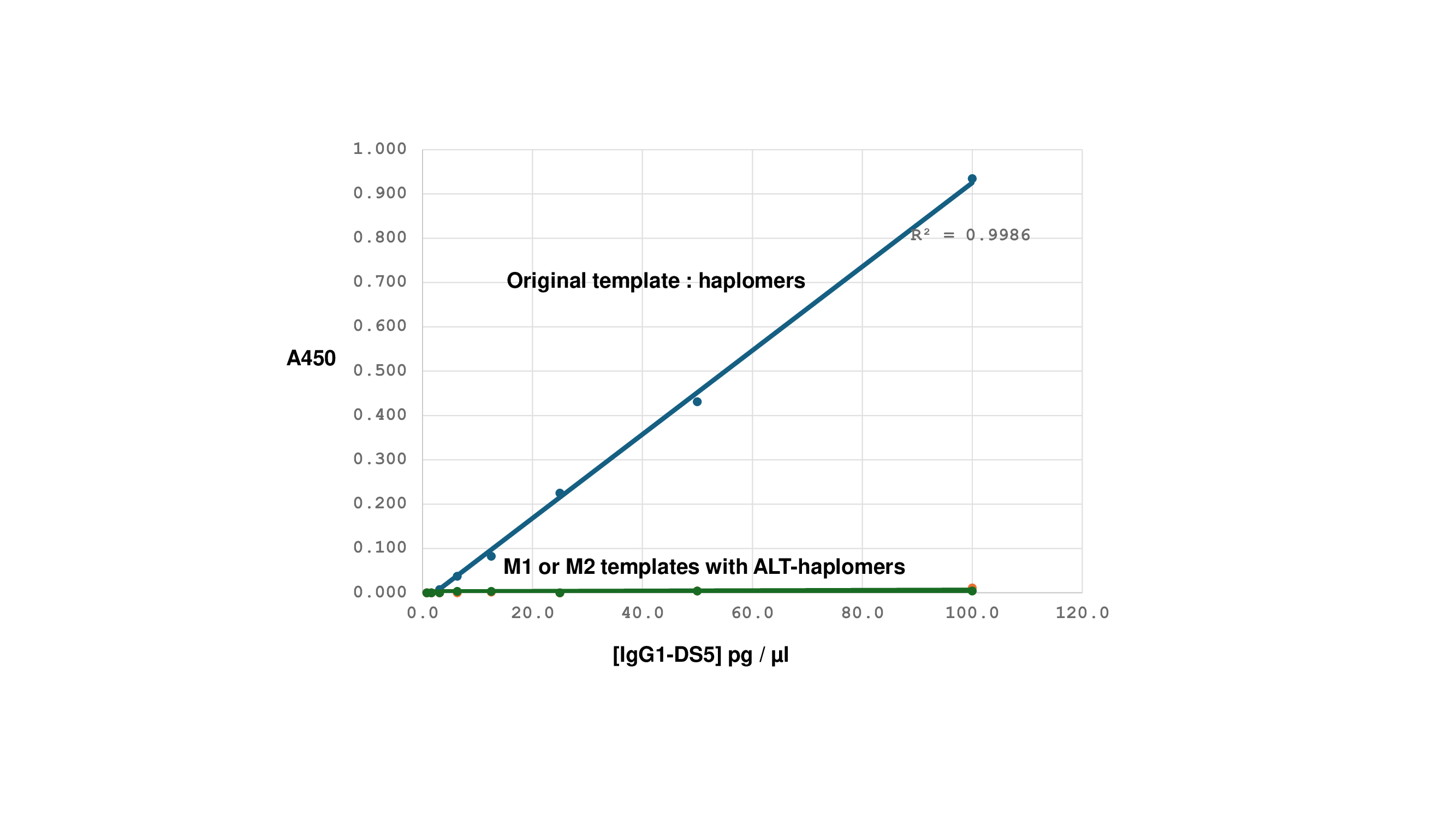

Supplement: Supplementary file 1 [file DataSheet1.zip › Supplementary Material.jpg/Figure S2.jpg]

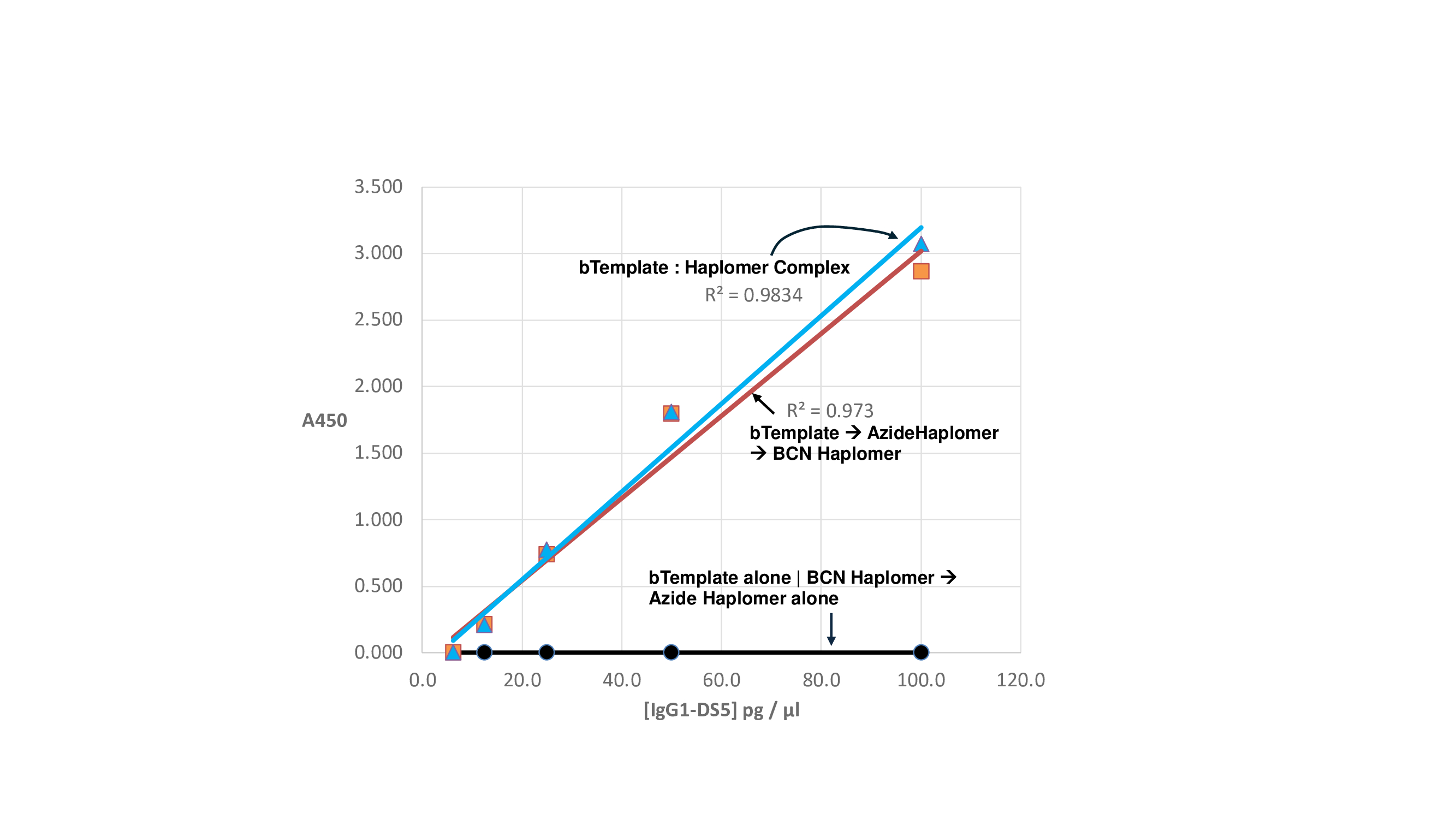

Supplement: Supplementary file 1 [file DataSheet1.zip › Supplementary Material.jpg/Figure S1.jpg]

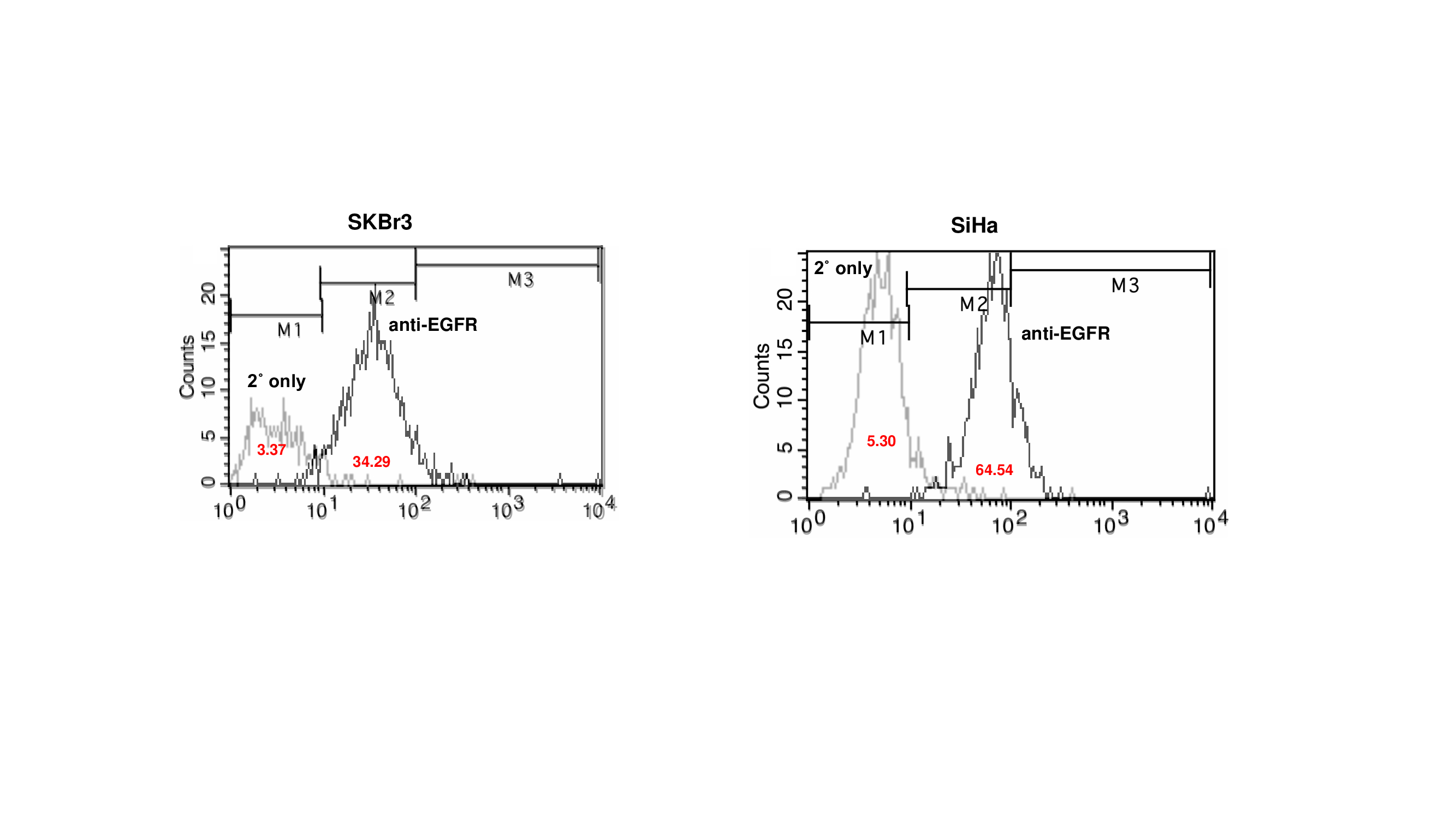

Supplement: Supplementary file 1 [file DataSheet1.zip › Supplementary Material.jpg/Figure S5.jpg]

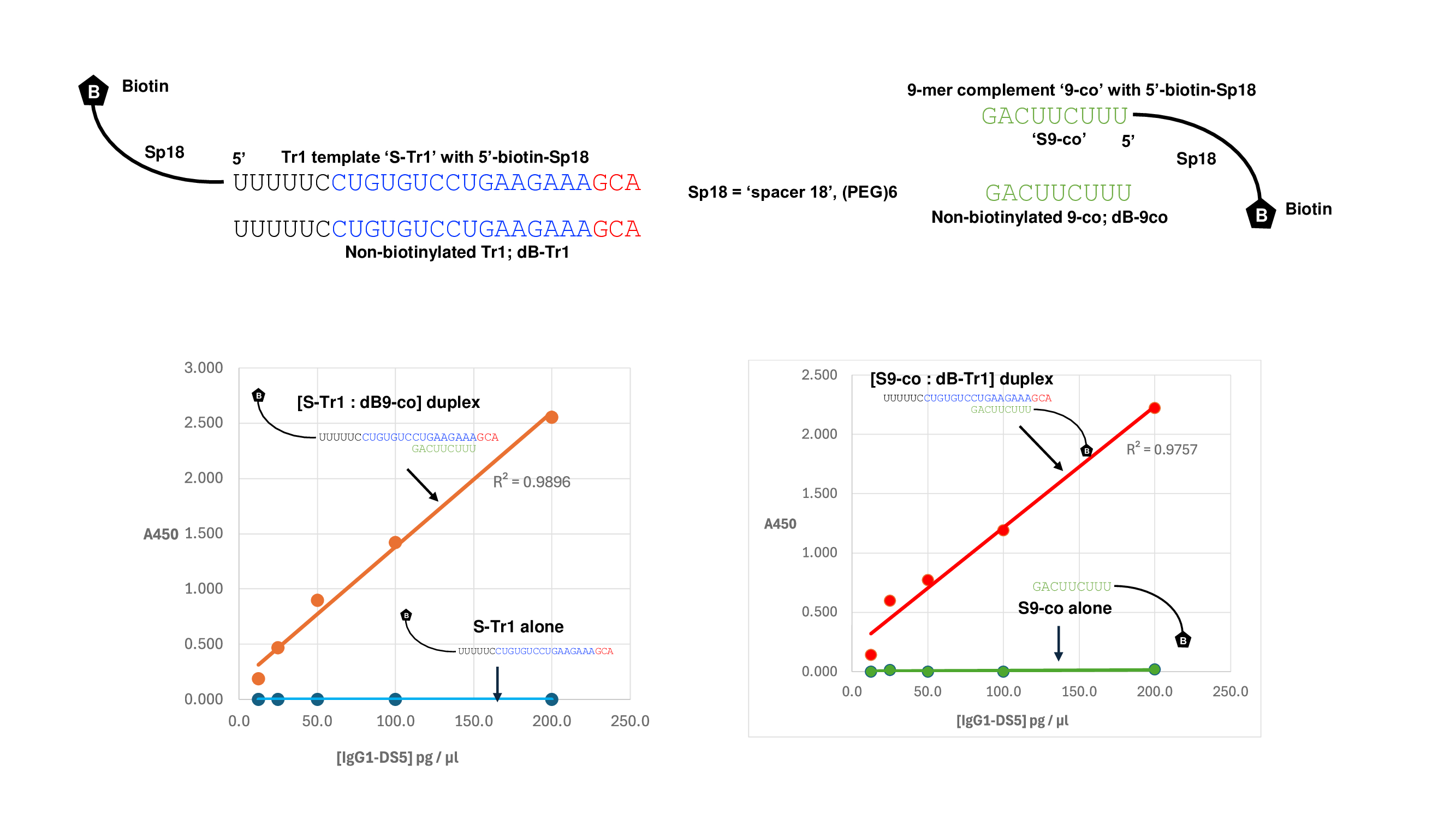

Supplement: Supplementary file 1 [file DataSheet1.zip › Supplementary Material.jpg/Figure S4.jpg]

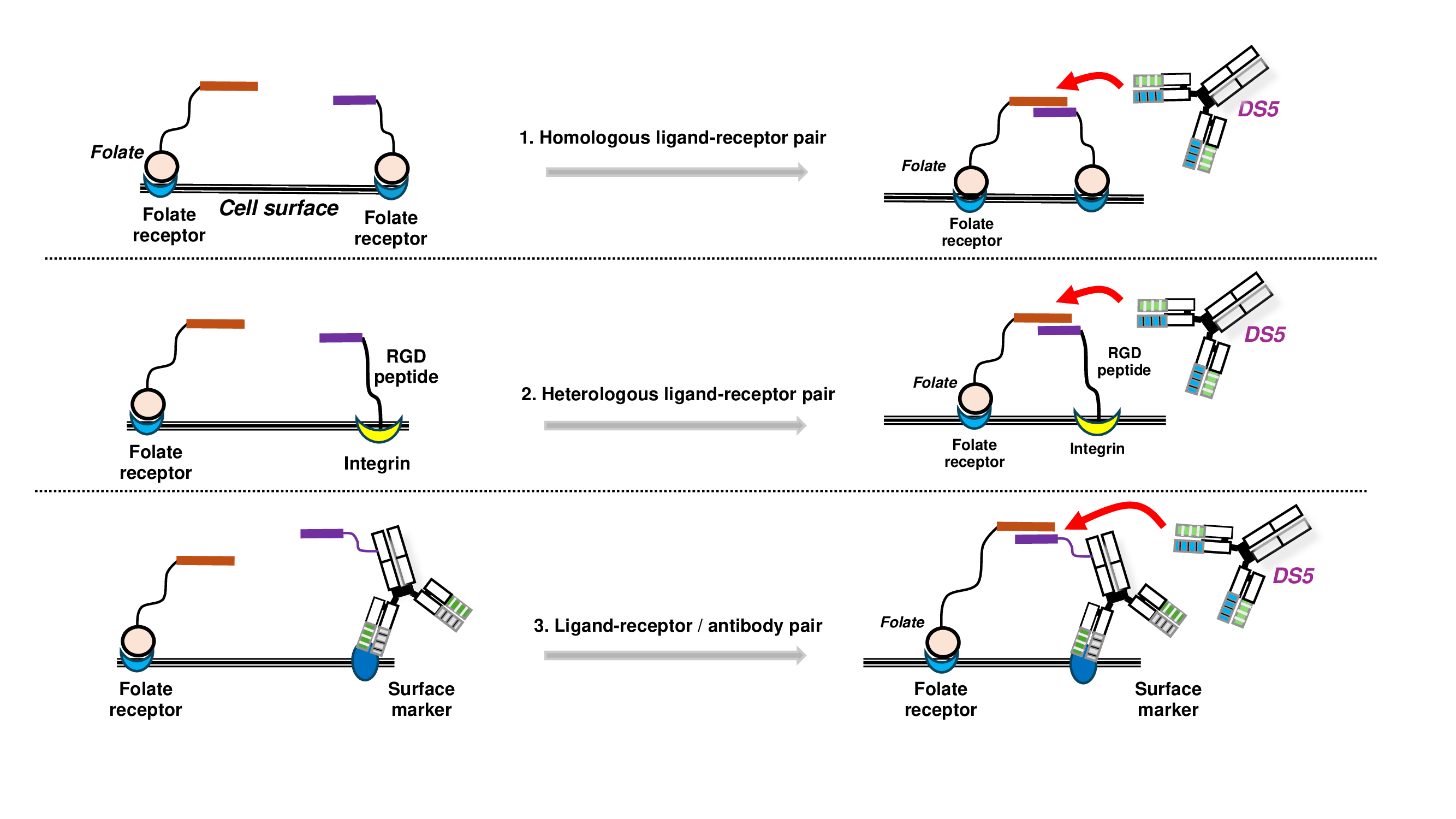

Supplement: Supplementary file 1 [file DataSheet1.zip › Supplementary Material.jpg/Figure S6.jpg]
